# Fully tunable on-chip meta-generator for multidimensional Poincaré sphere mapping

**DOI:** 10.1038/s41377-026-02364-9

**Published:** 2026-08-07

**Authors:** Shuang Zheng, Jing Luan, Tiange Wu, Liheng Yi, Zhenyu Wan, Qi Tian, Yuan Meng, Yijie Shen, Kaiyuan Wang, Liangjia Zong, Li Shen, Deming Liu, Ming Tang, Jian Wang, Minming Zhang

**Affiliations:** 1 School of Optical and Electronic Information and Wuhan National Laboratory for Optoelectronics, Huazhong University of Science and Technology, Wuhan, 430074 Hubei China; 2 National Engineering Research Center for Next Generation Internet Access System, Wuhan, 430074 Hubei China; 3 Optics Valley Laboratory, Wuhan, 430074 Hubei China; 4 Department of Mechanical Engineering and Materials Science, Washington University in St Louis, St Louis, 63130 MO USA; 5 Centre for Disruptive Photonic Technologies, School of Physical and Mathematical Sciences, Nanyang Technological University, Singapore, 637371 Singapore; 6 School of Electrical and Electronic Engineering, Nanyang Technological University, Singapore, 639798 Singapore

**Keywords:** Sub-wavelength optics, Silicon photonics

## Abstract

The angular momentum of light can be elegantly mapped onto high-order Poincaré spheres, providing a powerful framework for describing structured light beams. Such beams have shown extraordinary potential across diverse applications, including high-capacity optical communications, precision metrology, and quantum information processing. While various methods exist for generating structured light beams, the dynamic synthesis and flexible control of arbitrary vectorial states on diverse, multidimensional Poincaré spheres still rely on bulky free-space optical components, posing significant challenges for scalability and integration. To date, a fully tunable solution implemented on a single photonic chip has yet to be realized. Here, we present the first fully tunable on-chip meta-generator capable of dynamically mapping arbitrary scalar, vectorial, and hybrid modes across multiple Poincaré spheres, and extendable to higher-dimensional Poincaré hyperspheres within a four-dimensional Hilbert space. Our device is implemented on an eight-channel space-multiplexed multimode silicon photonic integrated circuit, where densely integrated mode multiplexers, amplitude–phase modulators, and an inverse-designed multimode meta-waveguide together enable compact, precise, and programmable control of structured light. The multimode meta-waveguide directly maps eight on-chip guided modes to orbital angular momentum (OAM), supporting broadband generation of high-purity OAM modes with diverse polarization states and topological charges. By simultaneously engineering amplitude, phase, polarization, and topological charge, we achieve full-field control over OAM mode bases, enabling fully tunable access to arbitrary scalar and vectorial states across more than eight distinct Poincaré spheres. This work represents a significant step toward reconfigurable on-chip manipulation of multidimensional Poincaré spheres, paving the way for advanced applications in optical communications, quantum photonics, and beyond.

## Abstract (web summary)

We demonstrate an integrated, fully tunable meta-generator that leverages an inverse-designed silicon meta-waveguide to dynamically synthesize and manipulate multidimensional structured light across multiple Poincaré spheres.

## Graphical abstract

**Figure:**
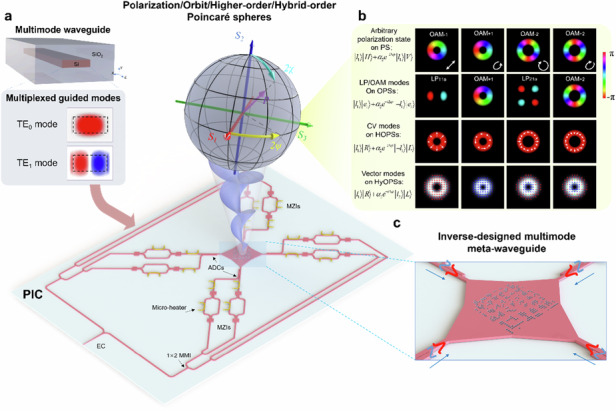

## Introduction

Light with circular polarizations and helical phase fronts, carries spin angular momentum (SAM) and orbital angular momentum (OAM) respectively, representing two fundamental properties of light waves and photons. These two degrees of freedom (DoFs) have recently been developed as an important toolbox for describing tailored structured light beams with unique transverse shapes^1,2^. The modern exploration of optical angular momentum indicates that optical vortex beams with helical phase fronts also carry OAM^3^. Beyond the optical phase vortices, another intriguing class of structured beams is the vectorial vortices, which are characterized by spatially varying polarization distributions or polarization singularities^4–6^. Notably, the SAM and OAM DoFs of cylindrical vector beams exhibit a physically non-separable nature, further enhancing their versatility and applications. The fundamental Poincaré sphere (PS) is commonly used to describe the polarization states of scalar light fields. Building upon this geometric representation, recent advancements have extended its framework to encompass more complex light structures. OAM modes, vector modes, and their intricate interconnections can now be mapped onto the surfaces of corresponding unit spheres^7–10^, including the OAM Poincaré sphere (OPS), the higher-order Poincaré sphere (HOPS), and the hybrid-order Poincaré sphere (HyOPS), providing a more comprehensive depiction of structured light fields. For instance, by replacing the orthogonal bases on the poles of the fundamental Poincaré sphere with OAM modes, the evolution between Laguerre-Gaussian (LG) and Hermite-Gaussian (HG) modes can be visualized on an analogous OPS^7^. The fundamental PS has also been extended to describe the higher-order polarization states of generalized vector vortex beam. By selecting different combinations of OAM charges for left- and right-circular polarization states at the sphere poles, more complex vector modes can be represented on modified spheres, such as the HOPS^8^ or the HyOPS^9^. On a HOPS, the two poles correspond to opposite OAM states with orthogonal circular polarizations, while the equator represents cylindrical vector (CV) beams, with special cases being the azimuthally and radially polarized light fields. Alternatively, when circularly polarized OAM states carry different topological charges (TCs), the resulting hybrid-order Poincaré sphere can describe full Poincaré beams or C-point singularities^10^. The unique phase and intensity distributions of these PS beams make them highly valuable for a broad range of applications, including mode-division multiplexing, optical micromanipulation, microscopy and optical metrology, to name just a few.

The dynamic and flexible generation of arbitrary structured light fields on diverse PSs is crucial for unlocking their full potential in various applications^11–19^. So far, the dynamic generation and manipulation of full PS beams has largely relied on free-space optical setups, such as spatial light modulators and lens arrays^20–23^. While many metasurface- and fiber-based schemes have been ingeniously designed and successfully demonstrated, they are still limited to generating a narrow range of structured beams and fall short in offering dynamic tunability and full reconfigurability^24–31^. Thanks to their advantages in miniaturization, versatility, and compatibility with complementary metal-oxide semiconductor (CMOS) technology, photonic integrated circuits (PICs) have emerged as a highly attractive platform for generating structured light beams. Promising approaches utilizing subwavelength structures on photonic integrated waveguides have been demonstrated to generate specific types of structured beams^32–43^. For instance, compact optical vortex emitters based on microring gratings have been proposed to extract light confined in whispering gallery modes, producing free-space beams with well-controlled OAM^33–36^. However, these microring resonant structures are limited to emitting fixed polarization state distributions with narrow optical bandwidth and cannot be tuned. More recently, a series of meta-waveguides combining various functional subwavelength photonic architectures with diverse waveguide platforms have demonstrated exceptional capabilities in controlling guided electromagnetic waves^37–40^. Driven by advanced inverse-design algorithms, these meta-waveguides can exhibit impressive performance with enhanced multifunctionality and ultracompact device footprint^41,42^. However, these devices are predominantly restricted to utilizing fundamental waveguide modes for the generation of linearly polarized OAM states, which inherently limits both the diversity and the number of accessible modal regimes. Furthermore, previous implementations lack the necessary degrees of freedom for dynamic manipulation, precluding the synthesis of more intricate and composite structure light fields. An alternative approach utilizing a programmable photonic mesh has also been demonstrated for the flexible and dynamic generation of structured light beams^43,44^. Nevertheless, the ability to dynamically generate arbitrary structured light beams across various PSs, including PS, OPS, HOPS, and HyOPS, remains an unresolved challenge. In this scenario, a laudable goal would be to develop an ultracompact and fully tunable PS beam generator on photonic integrated platform.

In this work, we propose and experimentally demonstrate a fully tunable Poincaré sphere beam generator implemented on a multimode silicon photonic integrated circuit. This device features a monolithic integration of four dual-mode multiplexers, an array of sixteen amplitude-phase modulators, and an inverse-designed multimode meta-waveguide. By synergizing high-order waveguide mode multiplexing with independent multi-channel addressing, the system enables the on-demand generation of arbitrary structured light beams across diverse PSs, including fundamental PS, OPS, HOPS, and HyOPS. Particularly, inspired by the mapping between OAM and other physical dimensions, such as spin, amplitude, frequency, and time^45–47^, we inversely designed a photonic-crystal-like (PhC-like) meta-waveguide to bridge the gap between on-chip guided modes and free-space OAM modes. This multimode meta-waveguide enables one-to-one conversion between eight space-multiplexed waveguide modes (TE_0_, TE_1_) from four directions and eight polarization- and charge-diverse OAM mode bases (*x*/*y*-polarized OAM_±1,±2_). Leveraging this orthogonal basis, arbitrary structured light fields on different PSs are flexibly synthesized and dynamically manipulated by orchestrating the amplitude and phase profiles of the on-chip guided modes. As the first single-chip solution capable of dynamically accessing all structured light states across more than eight distinct PSs, this work opens new avenues for advanced OAM generation and applications in the realm of integrated photonics.

## Results

### Multimode integrated silicon photonic circuit

**Fig. 1:**
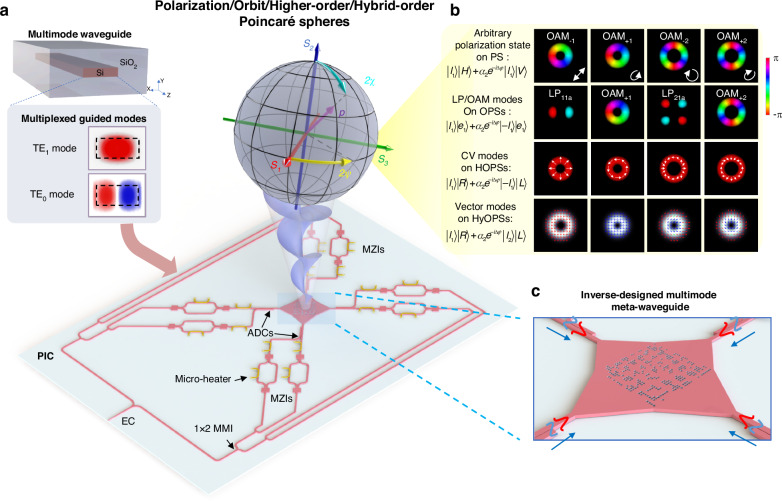
Device schematic diagram. **a** Schematic of fully tunable Poincaré sphere beam generator based on silicon photonic integrated circuits (not to scale). PIC photonic integrated circuits, EC edge coupler, ADC asymmetrical directional coupler for mode conversion and multiplexing, MZI thermo-optic (TO) Mach-Zehnder interferometer for amplitude modulation, MMI multimode interference coupler for optical power splitting, Multiplexed guided modes: Two typical waveguide modes (TE_0_ and TE_1_) for coupling to generate orbital angular momentum (OAM) beams. The simulated amplitude and phase distributions of the multiplexed waveguide modes before entering the meta-waveguide are shown in Fig. S1. **b** Mode field profiles of typical modes on PSs, OPSs, HOPSs, and HyOPSs. **c** Inversely-designed multimode meta-waveguide, featuring a subwavelength surface structure etched onto a 2D multimode silicon waveguide, facilitates the conversion of in-plane eight waveguide modes into off-chip OAM modes

Figure 1a illustrates the fully tunable Poincaré sphere beam generator based on an 8-channel multimode silicon photonic integrated circuit (PIC). The chip consists of four asymmetrical directional couplers (ADCs), eight tunable Mach-Zehnder interferometers (MZIs), eight phase modulators with thermo-optically (TO) microheaters, and an inversely-designed multimode meta-waveguide. This configuration enables the generation of all states across various PSs, as shown in Fig. 1b, including polarization PSs, OPSs, HOPSs, and HyOPSs. The light beam is first coupled into the chip through a high-efficiency edge coupler and is subsequently divided evenly into eight channels via a three-stage cascaded 1 × 2 splitter network. The relative amplitude and phase coefficients of the guided TE_0_ and TE_1_ modes in each channel are controlled using the on-chip amplitude and phase modulators, enabling programmable superpositions of the two modes. Following this, each ADC is used to multiplex fundamental transverse electric (TE_0_) mode and higher-order transverse electric (TE_1_) mode, allowing space multiplexing of four TE_0_ modes and four TE_1_ modes from four directions. The simulated eigenmode field distributions shown in Fig. 1a clearly reveal the significantly different electric field distribution characteristics of the TE_0_ and TE_1_ modes within the silicon waveguide. Detailed phase and magnitude distributions of the waveguide modes are provided in Fig. S1. The TE_0_ mode exhibits a simple optical field distribution, while the TE_1_ mode features a more complex pattern, with its electric field divided into two lobes of equal amplitude and opposite phase. The inversely-designed multimode subwavelength meta-structure embedded on the surface of silicon waveguide (Fig. 1c) functions as an 8-channel parallel mode converter, which can convert eight in-plane space-multiplexed guided modes (TE_0_, TE_1_) from four directions into eight polarization-/charge-diverse optical vortex modes (*x*/*y*-polarized OAM_±1, ±2_).

Notably, unlike our previous single-mode scheme, which is limited to the TE_0_ mode, the proposed multimode meta-waveguide enables the conversion of higher-order waveguide modes, accommodating both TE_0_ and TE_1_ modes for enhanced versatility and functionality. This advancement doubles the number of polarization- and charge-diverse OAM modes that can be generated compared to the single-mode approach^39^. By leveraging the rich and orthogonal OAM mode basis provided by the meta-waveguide, the space-multiplexed waveguide modes can be further transformed into a broad range of complex structured light beams through precise control of the mode profiles across the eight input channels—a level of flexibility and diversity unattainable with previous design. As illustrated in Fig. 1b, the proposed scheme enables the flexible generation of arbitrary polarization states on the fundamental PSs, arbitrary linearly polarized (LP) modes on OPSs, and arbitrary vector modes on HOPSs or HyOPSs. For example, as shown in the second row of Fig. 1b, LP modes exhibit uniform polarization distributions while possessing spatially varying amplitude and phase profiles, which can be synthesized by superimposing two OAM modes with the same polarization state but opposite TCs. CV modes, such as radially polarized (RP) and azimuthally polarized (AP) modes, exhibit spatially varying polarization states and can be generated by coherently combining two OAM modes with opposite TCs and circular polarizations. Detailed mapping relationships between waveguide modes and structured light beams (OAM, LP, and vector modes) are given in Supplementary Section S6 (see Fig. S7 and Table S1).

### Inverse-designed multimode meta-waveguide

**Fig. 2:**
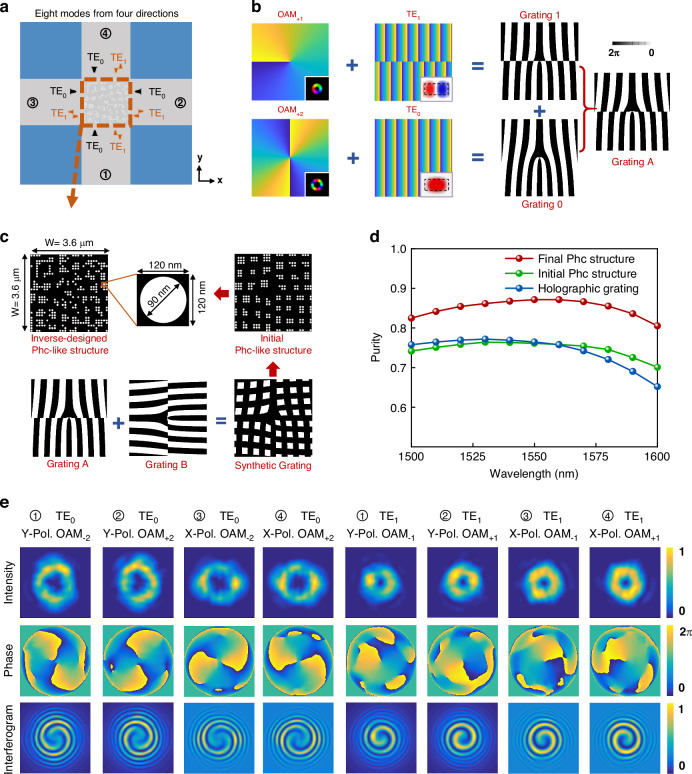
OAM mapping based on inversely-designed meta-waveguide. **a** 2D subwavelength surface structure region with four input ports and two space-multiplexed modes (TE_0_, TE_1_). **b** Illustration of holographic method producing superposed fork grating. The superposed Grating A is synthesized by Grating 1 and 0. Fork grating 1 and 0 is superimposed by the phase profiles of OAM_+1_ and TE_1_, and the phase profiles of OAM_+2_ and TE_0_, respectively. **c** Zoom-in view of the optimized photonic-crystal-like (PhC-like) structure with inverse design method. The final holographic grating is superimposed by Grating A and its 90-degree rotated pattern B. The initial PhC-like structure is then obtained by digitized binary of the final holographic grating. **d** Calculated average purities of eight OAM modes (*x*/*y*-pol. OAM_±1_, *x*/*y*-pol. OAM_±2_) before and after optimization over wide wavelength range (length w: 3.6 μm; depth h: 130 nm). **e** Simulated intensity profiles, phase distributions, and interferograms of the generated polarization- and charge-diversity OAM modes at 1550 nm

Figure 2a shows the schematic of the proposed inversely-designed multimode meta-waveguide, enabling the conversion between eight space-multiplexed guided modes (TE_0_, TE_1_) and eight polarization-/charge-diverse OAM modes (*x*/*y*-polarized OAM_±1_, _±2_). The design employs eight waveguide modes from four input ports. Due to the orthogonal polarization directions of the dominant electric fields in the TE_0_ and TE_1_ modes relative to the light propagation direction, *x*- or *y*-polarized OAM modes are generated when light propagates along the *y*- or *x*-direction, respectively. The sign and magnitude of the TC are determined by the propagation direction and the specific guided mode being excited. Figure 2b outlines the design process of the multimode meta-waveguide, which involves the formation and initialization of the holographic grating based on the principle of holographic superposition. The superposition of gratings can be selectively configured—it can be generated as shown in Fig. 2b, or by exchanging the correspondence between OAM modes and waveguide modes to produce a different grating pattern (see Supplementary Section S1). This is followed by the transformation of the initial PhC-like structure into the optimized final design through inversely-design techniques.

The formation, principle, and theory foundation of the initial surface grating on the silicon waveguide are based on the holographic method, which can be described as follows. The coupled interference between the emitted OAM mode (propagating vertically) and the in-plane guided TE_0_ mode (with the symmetric mode profile) gives rise to Grating 0 on the waveguide surface. The electric fields of the target OAM mode and the guided TE_0_ mode are expressed as:

1 $${E}_{{\rm{OAM}}}=O\exp (il\theta )$$

2 $${E}_{{{\rm{waveguide}}\_{\rm{TE}}}_{0}}={W}_{{{\rm{TE}}}_{0}}\exp (i{\beta }_{{{\rm{TE}}}_{0}}x)$$

where *O* and *W* are the amplitudes of the OAM mode and waveguide mode. *l* and $\theta ={\arctan }^{-1}$(*y*/*x*) represent the TC and azimuthal angle, respectively. ${\beta }_{{\mathrm{TE}}_{0}}$ is the propagation constant of the waveguide mode. The interference of the guided mode and the OAM mode form the holographic grating on top of the waveguide, which can be given as^39^:

3 $${H}_{{{\rm{grating}}\_{\rm{TE}}}_{0}}={|{E}_{{\rm{OAM}}}+{E}_{{{\rm{waveguide}}\_{\rm{TE}}}_{0}}|}^{2}={{O}_{{{\rm{TE}}}_{0}}}^{2}+{{W}_{{{\rm{TE}}}_{0}}}^{2}+2{O}_{{{\rm{TE}}}_{0}}{W}_{{{\rm{TE}}}_{0}}\,\cos ({\beta }_{{{\rm{TE}}}_{0}}x-{l}_{{{\rm{TE}}}_{0}}\theta )$$

To accommodate practical fabrication techniques, a simple binary function is employed to define the grating’s gray-scale distribution *H* (*x*, *y*). Specifically, *H* (*x*, *y*) = 0 when $\cos ({\beta }_{0}x-{l}_{0}\theta ) > 0$, and *H* (*x*, *y*) = 1 otherwise. Based on the principle of holography, the guided wave propagating in the +*x* direction (from left to right) interacts with Grating 0 to generate a vortex beam with a TC of -2. Conversely, the guided wave propagating in the -*x* direction (from right to left) produces a vortex beam with a TC of +2.

For TE_1_ mode with the asymmetric mode profile, the electric field can be written as

4 $${E}_{{\mathrm{waveguide}\_\mathrm{TE}}_{1}}=\left\{\begin{array}{l}{W}_{{\mathrm{TE}}_{1}}\exp (i{\beta }_{{\mathrm{TE}}_{1}}x)\,(y < 0)\\ {W}_{{\mathrm{TE}}_{1}}\exp (i({\beta }_{{\mathrm{TE}}_{1}}x+\pi ))\,(y > 0)\end{array}\right.$$

The distribution of the holographic grating can be given as

5 $${H}_{{\mathrm{grating}\_\mathrm{TE}}_{1}}={|{E}_{\mathrm{OAM}}+{E}_{{\mathrm{waveguide}\_\mathrm{TE}}_{1}}|}^{2}=\left\{\begin{array}{l}{{O}_{{\mathrm{TE}}_{1}}}^{2}+{{W}_{{\mathrm{TE}}_{1}}}^{2}+2O{W}_{{\mathrm{TE}}_{1}}\,\cos ({\beta }_{{\mathrm{TE}}_{1}}x-{l}_{{\mathrm{TE}}_{1}}\theta )\\ {{O}_{{\mathrm{TE}}_{1}}}^{2}+{{W}_{{\mathrm{TE}}_{1}}}^{2}+2O{W}_{{\mathrm{TE}}_{1}}\,\cos ({\beta }_{{\mathrm{TE}}_{1}}x-{l}_{{\mathrm{TE}}_{1}}\theta +\pi )\end{array}\right.$$

Using the design principles of Grating 0, Grating 1 is developed to generate OAM_±1_ modes when excited by TE_1_ modes propagating in the +*x* and -*x* direction, respectively.

It is important to note that for the strip waveguide with width of *W* = 3.6 μm, TE_0_ and TE_1_ modes have almost the same propagation constant $${\beta }_{eff}$$$$\,\approx$$11.4 μm^−1^(see Supplementary Section S2). To take advantage of mode multiplexing, Grating 0 and Grating 1 could be combined to form a new pattern Grating A, supporting multimode-to-OAM mapping. Thus, *y*-pol. OAM_-1_, *y*-pol. OAM_+1_, *y*-pol. OAM_-2_ and *y*-pol. OAM_+2_ modes could be generated when TE_1_ mode (along +*x* direction), TE_1_ mode (along -*x* direction), TE_0_ mode (along +*x* direction) and TE_0_ mode (along -*x* direction) are input. Similarly, Grating B can be generated by coupled interference between the vertically incident OAM modes (*x*-pol. OAM) and the in-plane guided modes (TE modes propagating along *y* direction). *x*-pol. OAM_-1_, *x*-pol. OAM_+1_, *x*-pol. OAM_-2_ and *x*-pol. OAM_+2_ modes can be converted by TE_1_ mode (along -*y* direction), TE_1_ mode (along +*y* direction), TE_0_ mode (along -*y* direction) and TE_0_ mode (along +*y* direction), respectively.

2D holographic grating can be synthesized by the superposition of grating A and B shown in Fig. 2c. Then, the 2D holographic grating is discretized as a PhC-like array composed of 30 ×30 pixels with a footprint of 3.6×3.6 μm^2^. Each pixel has a size of 120 nm × 120 nm and the shape of each pixel is a square with a circular silicon/silica hole at its center. The hole has a radius of 45 nm. The etching depth (*h*) of the circular could affect the OAM scattering efficiency and mode purity (Fig. S4). The initial PhC-like pixel array structure (at fixed *h* = 130 nm) can achieve average purity of more than 0.7 over wide wavelength range (1500–1600 nm, see Fig. S4e). The simulated far-field distributions for both 2D holographic grating and the initial PhC-like structure are shown in Figure S5 (see Supplementary Section S4). The electric field distributions for the generated OAM modes are not complete due to imperfect mode conversion. The initial PhC-like structure is discretized by digitized binary of the final holographic grating and then optimized by the DBS inverse design algorithm (Fig. S3). Each pixel is filled by silicon or silica, corresponding to the logical “1” or “0” state respectively. The PhC-like subwavelength pixels can eliminate the random change of patterns of air holes and offer an outstanding RIE-lag-insensitive feature^48,49^. The goal of our design is to obtain the optimized circular hole combinations (logical state arrangement) in the inverse design region to realize the maximum average phase purity of 8 emitted OAM modes. The mode purity is calculated based on the overlap integral between the generated field and the ideal target mode (as detailed in Methods)^39^. Consequently, the figure of merit (FOM) for the inverse design is defined as the average purity of these eight modes

6 $$FOM=\frac{1}{8}\sum ({P}_{y,+1}+{P}_{y,+2}+{P}_{y,-1}+{P}_{y,-2}+{P}_{x,+1}+{P}_{x,+2}+{P}_{x,-1}+{P}_{x,-2})$$

where *P*_*y*,+1_, *P*_*y*,+2_, *P*_*y*,-1_, *P*_*y*,-2_, *P*_*x*,+1_, *P*_*x*,+2_, *P*_*x*,-1_ and *P*_*x*,-2_ indicate the purity of vertically emitted *y*-Pol. OAM_+1_, *y*-Pol. OAM_+2_, *y*-Pol. OAM_-1_, *y*-Pol. OAM_-2_, *x*-Pol. OAM_+1_, *x*-Pol. OAM_+2_, *x*-Pol. OAM_-1_ and *x*-Pol. OAM_-2_ mode, respectively. For an ideal OAM emitter, the target FOM should be equal to 1. Figure. S3b (in Supplementary Section S3) indicates the optimized 2D pixel array pattern and calculated FOM values of initial PhC-like structure and two iterations, respectively. As can be seen, the FOM increases from 0.75 to 0.84 after two iterations.

As depicted in Fig. 2d, eight high-purity OAM modes are successfully generated across a broad wavelength range spanning 1500–1600 nm. At the central wavelength of 1550 nm, the average mode purity reaches a peak value of 0.871, representing a 14.6% improvement over the holographic grating and the initial PhC-like structure. Detailed simulations of the mode purities are provided in Fig. S4d–f (see Supplementary Section S4).

The far-field intensity profiles, phase distributions, collinear interferograms of eight polarization- and charge-diversity OAM modes at 1550 nm are numerically simulated. To provide a clear visualization of the mapping relationship, the evolution from in-plane guided modes to out-of-plane OAM modes is illustrated in Fig. S6 (see Supplementary Section S5). As shown in Fig. 2e, eight distinct polarization-/charge-diverse OAM modes are generated from on-chip guided modes, including *x*-Pol. OAM_-1_, *x*-Pol. OAM_+1_, *x*-Pol. OAM_-2_, *x*-Pol. OAM_+2_, *y*-Pol. OAM_-1_, *y*-Pol. OAM_+1_, *y*-Pol. OAM_-2_, and *y*-Pol. OAM_+2._ These modes are excited through specific guided mode-port configurations: TE_1_ mode (Port 3), TE_1_ mode (Port 4), TE_0_ mode (Port 3), TE_0_ mode (Port 4), TE_1_ mode (Port 1), TE_1_ mode (Port 2), TE_0_ mode (Port 1), TE_0_ mode (Port 2), respectively.

The intensity profiles exhibit characteristic annular structures, while the phase distributions reveal the expected spiral patterns. Specifically, the generated OAM_±1_ and OAM_±2_ modes demonstrate spiral phase distributions of 2π and 4π, respectively. The collinear interferograms further confirm the correct TCs, discernible through the counterclockwise or clockwise spiraling of the field intensity. Compared to the simulated results shown in Fig. S3 and Supplementary Section S4 for the holographic grating and the initial PhC-like structure, the far-field simulations in Fig. 2e exhibit more uniform electric field distributions. This improvement highlights an enhancement in mode purity achieved by the meta-waveguide design.

### Multidimensional Poincaré spheres mapping

The proposed device offers the capability for dynamic mapping of guided modes to complex structured beams. Leveraging the OAM mode basis provided by the 2D-patterned meta-waveguide, on-chip guided modes can be flexibly mapped to various structured light beams, including arbitrarily polarized OAM modes on fundamental PSs, linearly polarized (LP) modes on OPSs, cylindrical vector (CV) modes on HOPSs, and more complex vector modes on HyOPSs. These structured beams on different PSs can be defined as

7 $$|\psi |={\alpha }_{1}|{l}_{1}\rangle |{e}_{1}\rangle +{\alpha }_{2}{e}^{-i\Delta \varphi }|{l}_{2}\rangle |{e}_{2}\rangle$$

where *l*_1_ and *l*_2_ denote the OAM charges of the vortex beams with two scalar polarization states $\left|{e}_{1}\right\rangle$ and $\left|{e}_{2}\right\rangle$ respectively; the OAM charges and polarization states can be either identical or opposite, depending on the characteristics of the generated structured beams. ${\alpha }_{\mathrm{1,2}}$ and $\Delta \varphi$ correspond to the amplitudes of the two vortex beams and their relative phase, respectively.

**Fig. 3:**
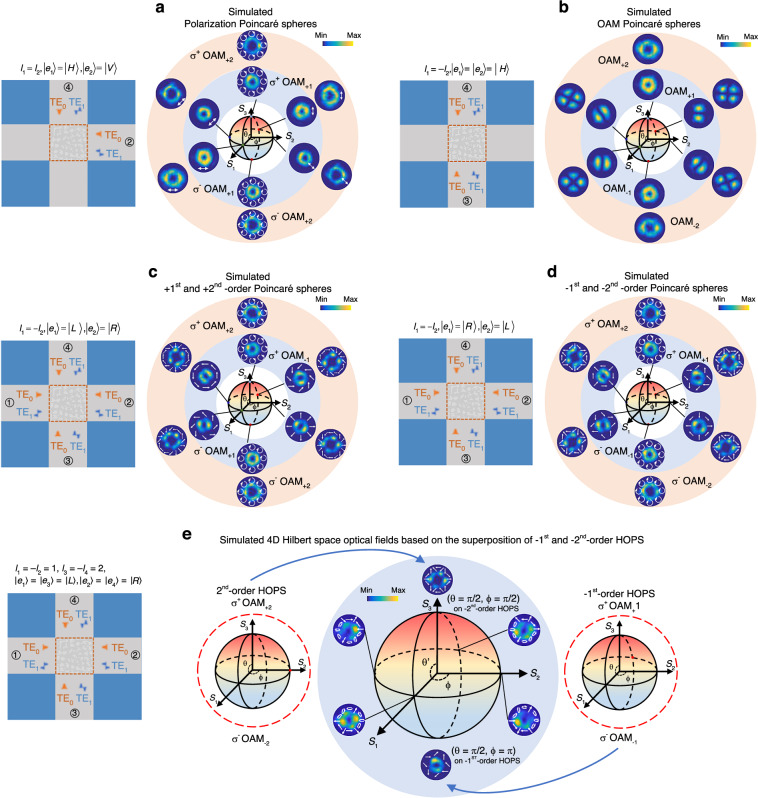
Controlled generation of high-dimensional vectorial states on different PSs. **a** Generating vortex modes, with arbitrary uniform polarization state on the fundamental polarization PSs. **b** Typical OAM and LP modes on OAM PSs. **c**, **d** Typical CV modes on four HOPSs. **e** Four-dimensional structured light via superposition of -1^st^ and -2^nd^-order HOPS

The scalar vortex beams OAM_+1,+2_ can be generated with arbitrary polarization state on the fundamental PS when *l*_1_ = *l*_2_ =+1/ + 2 (Figs. 1b and 3a). Any point on the PS can be expressed as a coherent superposition of two orthogonal linear polarization states $\left|{e}_{1}\right\rangle$ and $\left|{e}_{2}\right\rangle$. By controlling the amplitude and phase between two orthogonal linearly polarized OAM modes derived from guided modes, arbitrary polarized OAM modes on the PS can be synthesized. For instance, *x*-polarized OAM_+1_ mode is generated by the TE_1_ mode of Port 4, while *y*-polarized OAM_+1_ mode is derived from the TE_1_ mode of Port 2. The coherent superposition of these two modes can form a new OAM_+1_ mode with arbitrary scalar polarization states. Figure 3a shows the simulated optical fields for OAM_+1_ and OAM_+2_ modes, displaying six representative polarization states, including left- and right-circular polarization and four linear polarization states. These simulations confirm that the guided modes can be mapped to arbitrarily polarized OAM modes on the fundamental PS.

Similarly, an analogous OAM sphere is constructed to represent superpositions of left- and right-handed OAM modes, where the OAM charges satisfy *l*_1_ = -*l*_2_ =+1/ + 2, while maintaining an identical scalar polarization state. Any LP modes on OPSs can be generated by controlling the amplitude and phase profiles of the oppositely propagating guided modes. For example, *y*-polarized OAM_-1_ is generated by the TE_1_ mode of Port 1, while *y*-polarized OAM_+1_ originates from the TE_1_ mode of Port 3. By tuning the relative amplitude and phase values, the guided modes can be converted to arbitrary modes on the OPSs. As shown in Fig. 3b, the relative phase of the superposition dictates the orientation of LP modes on the equator. Similar results for OAM_±2_ superpositions are observed in the brown region of Fig. 3b.

Furthermore, the generation of first- and second-order CV beams can be achieved by controlling multiple guided modes. Figure 3c depicts two typical HOPSs, where the north and south poles represent left circularly polarized OAM_-*l*_ and right circularly polarized OAM_+*l*_, respectively. To synthesize a first-order CV beam on the HOPS, the phase relationship among four orthogonal OAM modes (*x*-OAM_±1_, *y*-OAM_±1_) must be precisely adjusted (see Supplementary Section S6). Detailed mapping relationships for CV modes are provided in Supplementary Table S1. In the inner blue region of Fig. 3c, first-order CV beams with cylindrical symmetry in both polarization and phase distributions are located on the equator, including radially and azimuthally polarized beams. These beams exhibit circular intensity profiles and spatially anisotropic polarization distributions (see Supplementary Fig. S8). The outer brown region represents second-order CV beams synthesized by four orthogonal OAM modes (*x*-OAM_±2_, *y*-OAM_±2_). Simulated electric field components and corresponding phase distributions (see Supplementary Fig. S9) confirm that the polarization distributions align with theoretical predictions. Two opposite HOPSs (-1^st^ and -2^nd^-order Poincare spheres) can be also achieved by controlling the same guided modes. Specifically, as shown in Fig. 3d, the north and south poles correspond to left circularly polarized OAM_+1_ and right circularly polarized OAM_-1_, respectively, while the CV beams located on the equator exhibit distinct polarization and phase distributions—consistent with the structural logic of HOPS but with reversed TC signs. More complex vector beams on the HyOPS can be realized by superimposing two scalar OAM modes with *l*_1_ ≠ -*l*_2_, while ensuring opposite circular polarization states.

Figure 3e illustrates the synthesis of a higher-dimensional structured light field by coherently superposing two orthogonal HOPS bases. The resulting optical field is expressed as a superposition of four constituent basis states^50,51^

8 $$|\psi \rangle ={\alpha }_{1}{e}^{-i{\varphi }_{1}}|{l}_{1}\rangle |{e}_{1}\rangle +{\alpha }_{2}{e}^{-i{\varphi }_{2}}|{l}_{2}\rangle |{e}_{2}\rangle +{\alpha }_{3}{e}^{-i{\varphi }_{3}}|{l}_{3}\rangle |{e}_{3}\rangle +{\alpha }_{4}{e}^{-i{\varphi }_{4}}|{l}_{4}\rangle |{e}_{4}\rangle$$

where *l*_1_, *l*_2_, *l*_3_, and *l*_4_ denote the OAM charges of four vortex beams with specific circular polarization states, $\left|{e}_{1}\right\rangle$, $\left|{e}_{2}\right\rangle$, $\left|{e}_{3}\right\rangle$, and $\left|{e}_{4}\right\rangle$, respectively. In this framework, two OAM modes, one with charge *l*_1_ and polarization state $\left|{e}_{1}\right\rangle$, another one with charge *l*_2_ and polarization state $\left|{e}_{2}\right\rangle$, are superposed to form a new composite basis, while another two OAM modes constitute a second basis. The parameters α_1_, α_2_, α_3_, and α_4_ represent the amplitudes of these four orthogonal constituent states. ${\varphi }_{1}$, ${\varphi }_{2}$, ${\varphi }_{3}$ and ${\varphi }_{4}$ denote the individual phases. By independently modulating the amplitude and phase of each channel, the platform enables meticulous control over the 4D superposition states, allowing for precise mapping within higher-dimensional Poincaré hypersphere. In Fig. 3e, the synthesized optical fields are constructed using four basis states with topological charges *l*_1_ = -*l*_2_ = 1 and *l*_3_ = -*l*_4_ = 2, paired with circular polarization components $\left|{e}_{1}\right\rangle$ = $\left|{e}_{3}\right\rangle$ = $\left|L\right\rangle$ and $\left|{e}_{2}\right\rangle$ = $\left|{e}_{4}\right\rangle$ = $\left|R\right\rangle$, respectively. The first composite basis, defined by the relation α_1_ = α_2_ and ${\varphi }_{1}-{\varphi }_{2}=\pi$, represents a state on the -1^st^-order HOPS and serves as the South Pole of higher-dimensional Poincaré hypersphere in Fig. 3e. The second composite basis follows α_3_ = α_4_ and ${\varphi }_{3}-{\varphi }_{4}=\pi /2$, corresponding to the -2^nd^-order HOPS and acting as the North Pole. By modulating the inter-basis phase difference ${\varDelta \varphi }_{{in}}=\left({\varphi }_{1},{\varphi }_{2}\right)-\left({\varphi }_{3},{\varphi }_{4}\right)$, four representative optical fields situated on the equatorial plane of the resulting superposition sphere can be generated.

By modulating the relative intensities and phases of the two basis modes, specifically through the independent control of the eight tuning channels, we can synthesize optical fields at any arbitrary position on the four-dimensional Poincaré sphere illustrated in Fig. 3e. Here, we showcase the field distributions at several representative locations on the sphere’s equatorial plane, which can be interconverted by precisely adjusting the phase difference between the two basis states. The decomposed polarization components of the simulated field substantiate the anticipated spatial polarization characteristics, with detailed results provided in Supplementary Fig. S10.

### Chip fabrication and measurement

**Fig. 4:**
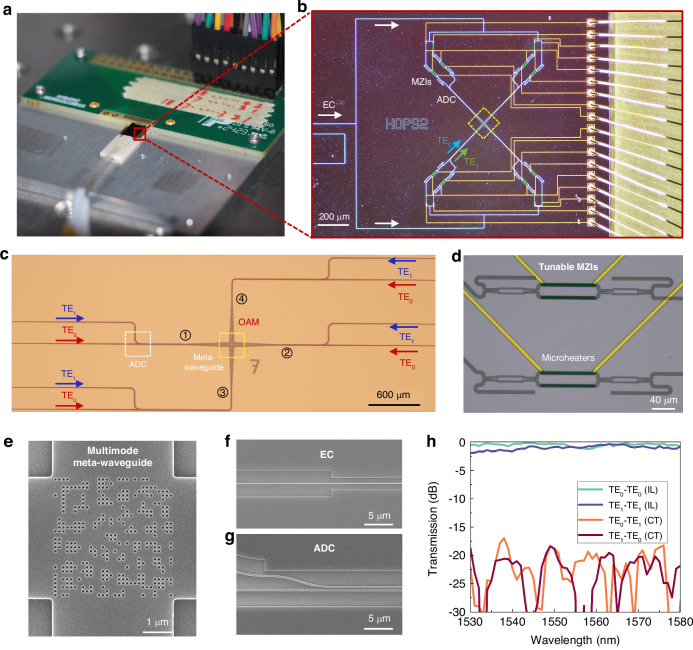
Device fabrication and characterization. **a** Microscopic image of the fully packaged chip, and **b** the fabricated silicon chip. **c**, **d** Partial close-up images of the chip. **e** SEM image of the inverse-designed meta-waveguide with PhC-like pixelated pattern. **f**, **g** SEM image of the EC and ADC. **h** The measured transmission spectra of the ADC(insertion loss(IL) and crosstalk(CT))

The chip is fabricated using a standard 220 nm thick silicon photonic platform. Detailed information on the fabrication process and characterization is provided in Supplementary Section S8 (see Fig. S11). The fully packaged chip, integrated with a thermos-electric cooler (TEC), is shown in Fig. 1a. All tuning components are wire-bonded to a carrier printed circuit board (PCB), and optical input/output is achieved using optical fiber arrays. The eighteen electrical tuning elements are controlled by a programmable control circuit (PCC) to enable dynamic reconfiguration. Figure 4b presents microscope images of the fabricated chip, showcasing the integration of an edge coupler, three-stage cascaded 1 × 2 splitters, eight TO MZIs, sixteen microheaters, four ADCs, and an inverse-designed meta-waveguide. Partial close-up images of the chip are provided in Fig. 4c, d. Figure 4e shows a scanning electron microscope (SEM) image of the inverse-designed meta-waveguide, revealing a PhC-like pixelated pattern that matches the optimized design shown in Fig. 2c. Additional SEM images of the edge waveguide coupler and the ADC are shown in Fig. 4f, g. The edge coupler facilitates efficient fiber-to-chip coupling, while the ADC is responsible for (de)multiplexing the TE_0_ and TE_1_ modes. The transmission spectra of the fabricated ADC, shown in Fig. 4h, demonstrate low insertion loss and crosstalk (< −20 dB) for both TE_0_ and TE_1_ modes, verifying the high-performance operation of the device. The total insertion loss of the system is approximately 18.3 dB, which comprises the following key components: 3 dB from the edge coupler, 0.5 dB from the three-stage Y-splitter, 0.8 dB from the tunable Mach-Zehnder interferometer (MZI), 1 dB from the mode multiplexer, and 13 dB from the meta-waveguide.

**Fig. 5:**
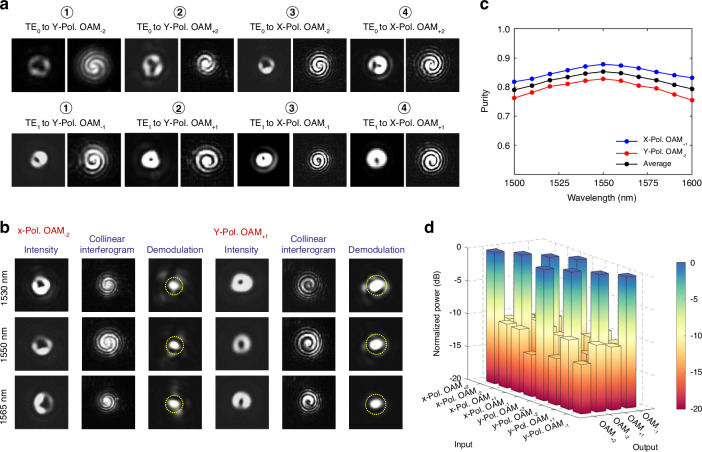
Experimental measurements of eight polarization- and charge-diversity OAM modes. **a** Measured far-field intensity profiles and collinear interferograms of the generated eight polarization- and charge-diversity OAM modes. **b** Measured far-field intensity profiles, collinear interferograms, and optical field demodulated via a SLM of the generated *x*-pol. OAM_-2_ and *y*-pol. OAM_+1_ modes at 1530, 1550, and 1565 nm for the meta-waveguide. **c** Optical field purity spectrum (1500–1600 nm) for the meta-waveguide. Results are shown for the generated *x*-pol. OAM_-2_ mode, *y*-pol. OAM_+1_ mode, and the average purity across all eight supported modes. **d** Measured histograms of the 8 × 4 crosstalk matrix for the meta-waveguide

The experimental setup shown in Fig. S18 (see Supplementary Section S15) is used to characterize the performance of the fabricated chip. First, we measure the far-field intensity profiles and coaxial interferograms of eight polarization- and charge-diversity OAM modes (*x*-Pol. OAM_-1_, *x*-Pol. OAM_+1_, *x*-Pol. OAM_-2_, *x*-Pol. OAM_+2_, *y*-Pol. OAM_-1_, *y*-Pol. OAM_+1_, *y*-Pol. OAM_-2_, and *y*-Pol. OAM_+2_) at 1550 nm. As shown in Fig. 5a, doughnut shaped intensity profiles and spiral interference patterns verify the helical phase fronts of the generated OAM modes. TC orders can be recognized by the number of spiral arms and the spiral direction in the interferograms, which are consistent with the simulation results. To verify the polarization state of the generated OAM modes, we rotate the polarizer before the camera to record the power variation of *x*-Pol. OAM_-2_ and *y*-Pol. OAM_+1_ modes to analyze the characteristic of the polarization (see Fig. S13 in Supplementary Section S9).

We also demonstrate the broadband generation of OAM modes from 1530 to 1560 nm. The measured far-field intensity profiles, collinear interferograms, optical field demodulated via a SLM of *x*-Pol. OAM_-2_ and *y*-Pol. OAM_+1_ modes are shown in Fig. 5b, respectively, at 1530, 1550 and 1565 nm. Hollow shaped intensity profiles, spiral collinear interference patterns, and Gaussian-type demodulated optical field reveal good performance of the generated OAM modes. The measured additional results for the meta-waveguide are shown in Fig. S12 (see Supplementary Section S9). To measure the mode crosstalk of the generated OAM modes, four kinds of phase patterns are selectively loaded onto the spatial light modulator (SLM) to demodulate the OAM_+ 1_, OAM_- 1_, OAM_+ 2_, OAM_- 2_ modes into Gaussian-like beam, respectively. As shown in Fig. S14 (see Supplementary Section S10), the emitted OAM modes of the meta-waveguide can be demodulated to a series of bright spots at the beam center (Gaussian-like beam) when meeting an inverse spiral phase pattern, while remains the doughnut field profiles with other TCs in the case of non-corresponding demodulation phase patterns. Figure 5c depicts the experimentally measured mode purity of the demodulated optical fields for the *x*-Pol. OAM_+1_ and *y*-Pol. OAM_-2_ modes, along with the average mode purity derived from all demodulated channels. Figure 5d shows the recorded crosstalk matrices, which can evaluate the influence on each other for the generation of polarization- and charge-diversity OAM modes for the multimode meta-waveguide at 1550 nm. As can be seen from the measured histograms, the average crosstalk values are less than −10 dB. This crosstalk primarily originates from fabrication imperfections in the etching process. Variations in etched aperture diameter and etching depth directly contribute to fluctuations in inter-mode crosstalk levels (see Supplementary Section S8).

### Fully tunable generation of Poincaré sphere beams

**Fig. 6:**
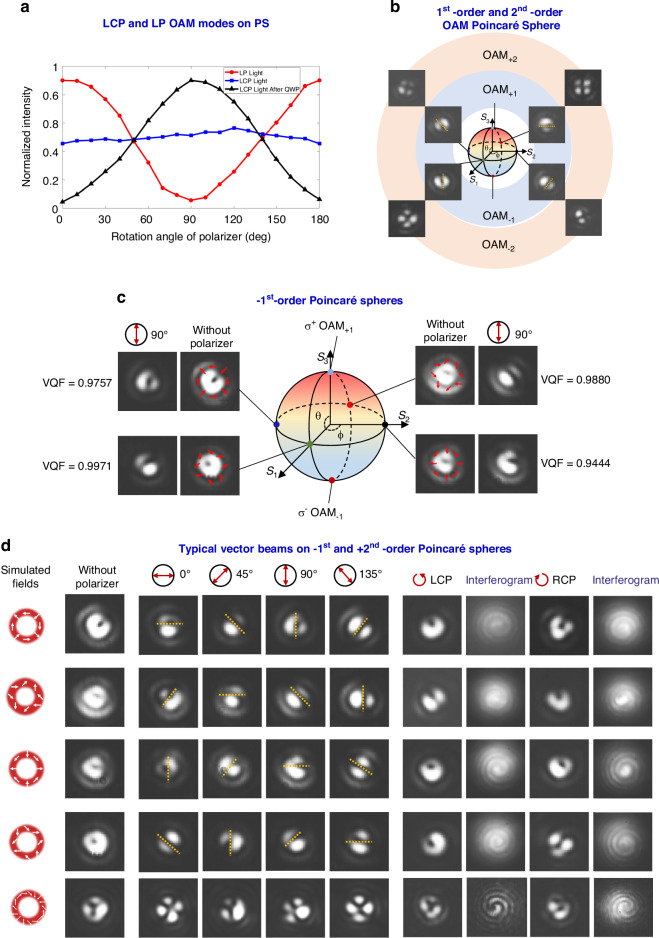
Tunable generation of diverse PS beams. **a** Normalized intensity profiles of typical OAM modes with LP, left-handed circularly polarized (LCP), and LCP states generated via a quarter-wave plate on OAM-based polarization PSs, measured after SLM demodulation. **b** Typical OAM and LP modes on two OAM PSs. **c** Typical vector modes on the -1^st^-order HOPS and their vector quality factors. **d** Typical vector modes on -1^st^ and +2^nd^-order HOPSs. Intensity profiles of the optical fields after transmission through a linear polarizer oriented at 0°, 45°, 90°, and 135° (denoted as *I*_0_, *I*_45_, *I*_90_, *I*_135_). The right- and left-handed circularly polarized components (*I*_R_, *I*_L_)(with their spiral collinear interference patterns) were obtained by passing the beam through a quarter-wave plate (QWP) followed by a linear polarizer oriented at +45° and –45°, respectively, relative to the fast axis of the QWP

The generated OAM mode basis can serve as a versatile platform for synthesizing all states across fundamental PSs, OPSs, and HOPSs and HyOPSs. For example, arbitrary polarization states on a fundamental Poincaré sphere can be generated by selecting two orthogonally polarized OAM modes (*x*-pol. OAM and *y*-pol. OAM), which can be derived from the guided modes in Ports 1 and 3 or Ports 2 and 4. To validate this capability, we experimentally demonstrate the generation of two representative polarized OAM beams. Figure 6a plots the normalized intensity profiles versus the polarizer rotation angle, measured after demodulation through a liquid crystal SLM. The sinusoidal blue curve corresponds to a 45°-polarized linearly polarized OAM_-1_ beam, confirming its successful generation. Similarly, the red curve represents the normalized intensity as a function of the polarizer angle for a left-handed circularly polarized (LCP) OAM_-1_ beam, illustrating precise control over polarization states. These results confirm that the fabricated device can generate OAM_±1_ and OAM_±2_ modes with arbitrary uniform polarization states across the fundamental PSs.

The fabricated chip can also generate arbitrary states on analogous OAM spheres. Distinctive from fundamental PSs, the poles of the OAM spheres correspond to two OAM modes with identical polarization states but opposite TCs, such as OAM_+1_ and OAM_-1_. In the experiment, *y*-polarized OAM_-1_ is converted from the TE_1_ mode of Port 1, and *y*-polarized OAM_+1_ is derived from the TE_1_ mode of Port 3, while the other six channels are deactivated by tuning the MZIs. By adjusting the TO phase shifters of these two channels, eight typical LP modes on the 1^st^ and 2^nd^-order OAM Poincaré spheres are generated in Fig. 6b. As shown in Fig. 3b, the relative phase of the superimposed modes determines the orientation of the LP modes along the equator. Similar results for the superposition of OAM_±2_ modes are also observed, demonstrating the flexibility of the device in generating arbitrary states on OAM PSs.

The experiment further investigates the generation of cylindrical vector (CV) beams with diverse polarization configurations. As illustrated in Fig. 6c, four representative CV states situated on the equatorial plane of the Poincaré sphere are characterized, corresponding to polarization orientation angles of *φ*_0_ = 0°, 45°, 90°, and 135°. Upon passing through a vertically oriented linear polarizer, the characteristic doughnut-shaped intensity profiles are resolved into distinct two-lobed patterns. The azimuthal orientation of the resulting nodal lines (dark fringes) provides unambiguous confirmation of the polarization topology and the specific states of the synthesized CV beams. We calculated the vector quality factor (VQF) of the generated vector beams via a basis-independent method using Stokes parameters^52^. With this method, the vector quality factors (VQF) for the vector beams in Fig. 6c are calculated as 0.9757, 0.9880, 0.9444, and 0.9971, respectively, based on the optical field distributions shown in Fig. 6d.

The chip also demonstrates the capability to flexibly generate complex CV beams on different HOPSs. As shown in Fig. 6d, the chip is employed to emit typical vector modes on the -1^st^ and +2^nd^-order HOPSs. The first row illustrates the measured intensity profiles of a vector mode located on the equator of -1st-order HOPS, where a polarizer with varying orientation angle is used to verify the polarization state of the emitted light fields. When the light passes through the polarizer with orientation angle of 0° and 90°, two bright spots appear along the vertical and horizontal directions, respectively. A similar phenomenon is observed for polarizer orientation angles of 45° and 135°, confirming that the measured polarization distributions align well with the target polarized CV beam. To further validate the polarization state of generated CV beam, circularly polarized Gaussian beams are coaxially interfered with the light fields. A first-order CV beam can be described as a superposition of a vortex mode with RCP OAM_-1_ and a vortex mode with LCP OAM_+1_. When the generated light field is interfered with a reference LCP (RCP) Gaussian beam and analyzed through a circularly polarized filter composed of a quarter-wave plate (QWP) and a polarizer, the resulting interferograms exhibit helical interference fringes. These fringes verify the presence of LCP OAM_+1_ and RCP OAM_-1_ components, as shown in Fig. 6d. Additionally, the chip achieves the generation of a typical vector mode on the +2^nd^-order HOPS, featuring a more intricate polarization vector distribution. These results further showcase the versatility of the chip in generating structured light fields with complex polarization characteristics. It is worth noting that more than the states on the equator and longitude, any point on the surface of PSs can be achieved by adjusting the relative amplitude and phase difference of four input guided waves, despite of these specific cases adopted in this work for demonstration.

**Fig. 7:**
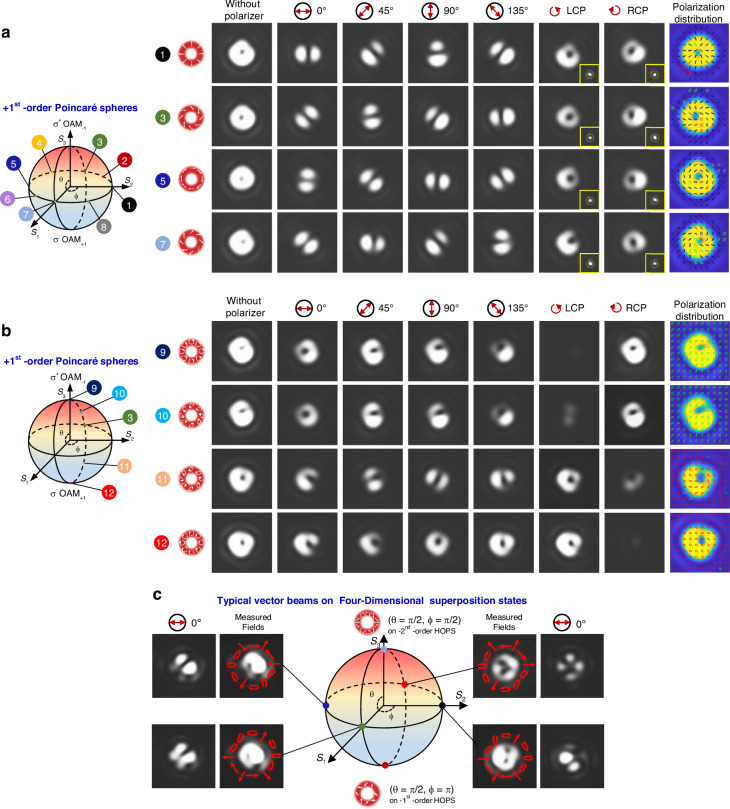
Fully tunable synthesis of vector beams on the +1^st^-order HOPS and 4D superposition states. **a** Evolution of the states on the equator of HOPS. **b** Evolution of the states on the longitude of HOPS. The figure presents a systematic analysis of the structured optical field: the first column shows the original field distribution; columns 2–5 display its intensity profiles after transmission through linear polarizers oriented at 0°, 45°, 90°, and 135°; columns 6–7 present the right- and left-handed circularly polarized components (RCP, LCP) along with their corresponding SLM-demodulated Gaussian-like beam, obtained by passing the beam through a quarter-wave plate followed by a linear polarizer oriented at +45° and –45°, respectively, relative to the QWP’s fast axis; and column 8 shows the reconstructed polarization map, derived from the component fields illustrated in the preceding columns. **c** Typical vector modes of four-dimensional optical fields based on the superposition of -1^st^ and -2^nd^-order HOPS

Figure 7 demonstrates the fully tunable synthesis of the device across the +1^st^-order HOPS. Figure 7a presents the optical fields at four representative equatorial positions on the +1^st^-order HOPS. For each position, the corresponding optical field is characterized through a series of measurements: the original intensity profile, its projections along four linear polarization directions, left- and right-handed circular polarization components extracted via a quarter-wave plate (along with their respective demodulated patterns), and the reconstructed full-Stokes polarization distribution. The detailed field-component data for equatorial positions 2, 4, 6, and 8 are provided in Supplementary Fig. S15 (see Supplementary Section S11). The experimental results verify that the polarization distribution of the beam generated by this device is in excellent agreement with the theoretical expectation. Moreover, the well-defined left- and right-handed circular polarization components and their corresponding demodulated field distributions further confirm the high structural purity of the generated optical field.

Figure 7b illustrates representative optical fields sampled along a specific longitude of the +1^st^-order HOPS, where the state at position 3 is identical to its counterpart in Fig. 7a. This sequence provides a clear visualization of the continuous and seamless tuning along the meridian: the states evolve from pure circular polarization at the poles through elliptical configurations with increasingly pronounced vectorial features, ultimately culminating in linearly polarized vector beams at the equatorial plane. While the results in Fig. 7 are obtained from the +1^st^-order HOPS, the same fully tunable behavior is experimentally demonstrated across all Poincaré spheres presented in Fig. 3, confirming the device’s capability for multidimensional optical field reconfiguration. Experimental demonstrations of continuous tuning along the HOPS equatorial and meridional planes are presented in Supplementary Videos S2 and S3. These videos capture the continuous evolution of mode profiles, effectively illustrating the device’s dynamic tuning performance.

By adopting vector beams from the –1^st^-order and -2^nd^-order HOPS as two orthogonal basis states, optical fields residing in a four-dimensional superposition states can be synthesized, as illustrated in Fig. 7c. Arbitrary states on the resulting Poincaré sphere are accessible by tuning the relative phase and intensity ratio between the two bases. The optical fields presented in the figure all lie on the equatorial plane of this sphere and are obtained by varying the inter-basis phase difference. The experimentally synthesized fields and their horizontally polarized components exhibit excellent agreement with the corresponding ideal simulations, underscoring the device’s high performance in multi-dimensional optical-field control. Furthermore, by shifting the coordinates of the two basis states on the HOPS, we can reconfigure the specific sphere associated with the 4D superposition states. Experimental results demonstrating 4D field synthesis using distinct sets of basis states are provided in Section S12 of the Supplementary Material.

## Discussion

In summary, we have successfully demonstrated a fully tunable Poincaré sphere beam generator on a silicon photonic chip. The device integrates an edge coupler, three-stage cascaded 1 × 2 splitters, eight tunable MZIs with TO phase shifters, sixteen microheaters, four ADCs, and an inversely-designed multimode meta-waveguide. Notably, leveraging the multimode meta-waveguide for eight-channel mode conversion and eight-channel amplitude-phase modulation, our platform enables dynamic generation of structured light beams spanning more than eight distinct PSs. Experimentally, we have demonstrated the versatility of the device by generating a range of structured beams, including arbitrary polarization states on the fundamental PS, four LP modes on the first- and second-order OAM PSs, and CV modes on the first- and second-order HOPSs. The successful realization of these diverse structured light beams validates our chip design and implementation, paving the way for high-performance and reconfigurable photonic integrated circuits for practical applications.

Compared to existing devices, this work advances the state of the art in two key aspects. First, it innovatively employs higher-order waveguide mode (TE_1_) and utilizes inverse-designed subwavelength structure to achieve high-purity OAM beam emission corresponding to each incident waveguide mode. Unlike previous devices confined to fundamental-mode operation, our design successfully incorporates multiple high-order waveguide modes into the system through accurate modal field matching, thereby substantially expanding the dimensionality of tunable optical modes. Furthermore, the device exhibits remarkable reconfigurability and programmability. Utilizing eight independent optical channels capable of simultaneous phase and amplitude control, we demonstrate the flexible synthesis of arbitrarily complex polarization and phase-structured optical fields across multiple Poincaré spheres and 4D superposition states. This architecture establishes a novel integrated photonic platform for versatile optical field manipulation. A comparison of our work with state-of-the-art on-chip structured light beam generators is summarized in Table S2 (see Supplementary Section S16). By leveraging an inversely-designed multimode meta-waveguide that supports both fundamental and higher-order modes, our approach facilitates broadband and efficient conversion between eight guided modes and eight orthogonal OAM modes. Further empowered by a tunable silicon photonic platform, our device facilitates the fully tunable generation of arbitrarily structured beams across more than eight distinct PSs, surpassing previously reported works in the number and variety of modes^28,37,39,40,42–44^.

Our existing platform uses two orthogonal waveguide arrays to enable multimode operation. By extending this architecture to include more intersecting channels (e.g., 6 or 8 channels), we can linearly scale the mode count. For instance, incorporating 8 input channels (each supporting 2 guided modes) would theoretically enable 16 spatial mode generation, provided the inverse-designed meta-waveguide is optimized to resolve the increased modal diversity. The integration density, particularly for mode directional couplers, can be significantly enhanced through targeted design optimizations: our current asymmetric directional couplers and tapered waveguide structures (each exceeding 200 μm in length) are primary contributors to device footprint. To miniaturize these components, we propose implementing inverse-designed compact mode converters. Thus, we compress the mode transition length to the 10 μm scale, achieving an order-of-magnitude reduction compared with conventional designs^53,54^. We also investigated the scalability by increasing the waveguide width and correspondingly enlarging the holographic grating region. The preliminary simulations suggest that a wider multimode waveguide could potentially support additional higher-order transverse electric (TE) modes (e.g., TE_2_, TE_3_), thereby expanding the available spatial mode basis. Meanwhile, extending the coverage area of the digitized holographic grating may increase the interaction region between the guided modes and the subwavelength structures, which could in turn possibly improve the diffraction efficiency and to some extent. We explored the approach of increasing waveguide width to enhance the diffraction efficiency of the device, as demonstrated in our simulation results (see Supplementary Section S18). The diffraction efficiency shows significant improvement as the grating area expands.

The measured rise time and fall time of the TO phase shifter are 21.4 μs and 22 μs, respectively (see Supplementary Section S20). This speed is primarily constrained by the inherent thermal dynamics of the TO effect. Replacing the TO phase shifters with PN junction phase shifters/LNOI-Si modulators can significantly improve the switching speed to nanosecond regime^55,56^. For example, PN junction phase shifters rely on the plasma dispersion effect, where carrier injection or extraction modulates the refractive index of silicon. This carrier-driven process operates on a nanosecond timescale, which is five orders of magnitude faster than TO mechanisms. These alternatives provide flexible pathways to meet the high-speed requirements of future communication and quantum photonic systems.

In our current workflow, we used the 3D FDTD simulations assisted with improved direct-binary-search (DBS) algorithm to design optimum pattern. It takes about 168 h to obtain the optimized pixel array of the meta-waveguide after two rounds of iteration, on the workstation with a twenty-core central processing unit (Intel Core i9-10900X). To reduce simulation time and optimization cycles, we have explored the potential of adjoint-method-based inverse design as a more efficient alternative. Preliminary benchmarks indicate that replacing DBS with the adjoint method could enhance optimization efficiency by approximately 5-fold. This improvement stems from the adjoint method’s ability to compute gradients of the objective function (e.g., mode conversion efficiency, phase matching) in a single simulation run, rather than requiring separate evaluations for each design parameter adjustment. The device footprint can be significantly reduced by leveraging inversely-designed mode-division multiplexed photonic circuits, enabling ultra-dense on-chip integrated PS beam generator^48,49,53,54^.

Currently, mainstream foundries (e.g., TSMC, SMIC) already offer mature processes supporting 90 nm-diameter hole fabrication via industrial-grade deep ultraviolet (DUV) lithography, which avoids the throughput limitations of electron-beam lithography (EBL) and is well-suited for large-scale wafer production. Moreover, as reported in previous report^54^, the hole diameter can be further scaled up to 130 nm (hole size: 130 nm in diameter; nanohole gap distance: 120 nm) while maintaining the meta-waveguide’s mode conversion performance. To further improve fabrication tolerance, an inverse design method called robust adjoint method could be also used to design fabrication-tolerant digital nanophotonic devices^53^.

The integration of on-chip laser sources would enhance both scalability and compactness, paving the way for fully integrated, high-performance structured light beam generation systems. Since our chip can simultaneously manipulate vector beams on two distinct PSs, it serves as a powerful tool for the reconfigurable and dynamic generation of optical skyrmions, providing a versatile platform for customizing topological light fields on demand^57–61^.

Finally, while this work demonstrates high-fidelity control over classical light, the underlying reconfigurable architecture offers a compelling pathway toward high-dimensional quantum photonics. The precise and fully tunable manipulation of the Poincaré sphere serves as a fundamental prerequisite for generating structured quantum states, such as high-dimensional entangled states. Recent advancements have successfully demonstrated on-chip single-photon generation of structured light^62–65^. By monolithically integrating quantum sources, such as spontaneous four-wave-mixing (SFWM) devices^65^, with our multimode meta-generator, our platform could facilitate the scalable synthesis and routing of multidimensional quantum states. Future research focusing on ultrafast electro-optic modulation and the on-chip integration of on-demand quantum emitters will be essential to translate this platform into a robust testbed for scalable quantum information processing^66^.

## Materials and methods

### Mode purity calculation

To quantitatively evaluate the quality of the generated structured light beams, we employed the overlap integral method. The mode purity is defined as

9 $$P=\frac{|\int {E}_{0}^{\ast }(x,y,z){E}_{t}(x,y,z)dxdydz{|}^{2}}{\int |{E}_{0}(x,y,z){|}^{2}dxdydz\int |{E}_{t}(x,y,z){|}^{2}dxdydz}$$

where *E*_0_(*x*, *y*, *z*) and *E*_*t*_(*x*, *y*, *z*) represent the complex electric field distributions of the generated beam and the ideal target mode, respectively. The maximum overlap value obtained through optimization procedure was adopted as the mode purity.

### Device simulation

The finite-difference time-domain (FDTD) method is used to perform the physical simulations of the meta-waveguide, ADC, splitter, and optical MZI. The finite element method (FEM) method is utilized to calculate the waveguide mode profiles.

### Chip fabrication and assembly process

As indicated in Fig. S11, the photonic chip is fabricated and experimentally demonstrated on standard 220 nm silicon-on-insulator (SOI) wafer. More details can be found in Supporting information, Section S8. The chip is mounted onto a copper chuck using thermal epoxy for efficient heat dissipation. Wire bonding is employed to establish electrical connections between the chip and the printed circuit board (PCB). Low-loss, broadband fiber-to-chip coupling is achieved via inverse-taper edge couplers, facilitating efficient optical interfacing. A fiber array with a 127 μm pitch is precisely aligned and fixed to the on-chip edge coupler array. To ensure thermal stability, a thermo-electric cooler (TEC) is integrated, maintaining the chip at a controlled ambient temperature.

### Polarization characterization and reconstruction

The spatially varying polarization distributions of the generated vector beams were reconstructed by measuring the full set of Stokes parameters (*S*_0_, *S*_1_, *S*_2_, *S*_3_). We recorded the intensity profiles of the optical field after passing through a linear polarizer oriented at angles of 0°, 45°, 90°, and 135°(*I*_0_, *I*_45_, *I*_90_, *I*_135_). Additionally, the right- and left-handed circularly polarized components (*I*_R_, *I*_L_) were obtained by passing the beam through a quarter-wave plate (QWP) followed by a linear polarizer oriented at +45° and −45° relative to the QWP’s fast axis, respectively. The Stokes parameters were then calculated as *S*_0_ = *I*_0_ + *I*_90_, *S*_1_ = *I*_0_ - *I*_90_, *S*_2_ = *I*_45_ - *I*_135_, and *S*_3_ = *I*_R_ - *I*_L_. Using these spatially resolved Stokes parameters, the polarization ellipticity and orientation angle at each point were retrieved to reconstruct the full polarization state distribution of the vector optical fields. The detailed methods for this polarization reconstruction are provided in Supplementary Section S13.

## Supplementary information

Supplementary_Video_S1_Broadband generation of vector beam

Supplementary_Video_S2_Continuous evolution of optical fields along the equator of the +1st-order HOPS

Supplementary_Video_S3_Continuous evolution of optical fields along the meridian of the +1st-order HOPS

Supplementary Materials of LSA20252643

## Data Availability

The data that support the findings of this study are included in the article and its supplementary information. Other data are available from the corresponding author upon request.
