# Supplementary material for: Fully tunable on-chip meta-generator for multidimensional Poincaré sphere mapping
Source: Light Sci Appl. 2026 Aug 7;15:341. doi: 10.1038/s41377-026-02364-9 (PMC13451280; doi:10.1038/s41377-026-02364-9)
Supplement: Supplementary file 1 [file 41377_2026_2364_MOESM4_ESM.docx]

Supplementary Materials for

**Fully Tunable On-Chip Meta-Generator for Multidimensional Poincaré Sphere mapping**

Shuang Zheng^1,2^^,3,*, +^, Jing Luan^1,2,+^, Tiange Wu^1,2,+^, Liheng Yi^1,2^, Zhenyu Wan^1^, Qi Tian^1^, Yuan Meng^4^, Yijie Shen^5,6^, Kaiyuan Wang^1,2^, Liangjia Zong^1,2,3^, Li Shen^1,3^, Deming Liu^1,2^, Ming Tang^1,2,3^, Jian Wang^1,3,*^ and Minming Zhang^1,2,3,*^

*^1^School of Optical and Electronic Information and Wuhan National Laboratory for Optoelectronics, Huazhong University of Science and Technology, Wuhan, 430074, Hubei, China*

*^2^National Engineering Research Center for Next Generation Internet Access System, Wuhan, 430074, Hubei, China*

*^3^Optics Valley Laboratory, Wuhan, 430074, Hubei ,China*

*^4^Department of Mechanical Engineering and Materials Science, Washington University in St Louis, St Louis, 63130, MO, USA*

*^5^Centre for Disruptive Photonic Technologies, School of Physical and Mathematical Sciences, Nanyang Technological University, Singapore, 637371, Singapore*

*^6^School of Electrical and Electronic Engineering, Nanyang Technological University, Singapore, 639798, Singapore*

*These authors contributed equally: Shuang Zheng, Jing Luan, Tiange Wu*

*** [*zshust@hust.edu.cn*](mailto:zshust@hust.edu.cn)*,* [*jwang@hust.edu.cn*](mailto:jwang@hust.edu.cn)*, and mmz@hust.edu.cn*

Content

[Section S1. The mapping order selection	3](#_Toc221191431)

[Section S2. Effective index and propagation constant	4](#_Toc221191432)

[Section S3. Details of simulation and optimization process	4](#_Toc221191433)

[Section S4. Simulation results of initial and optimized structures	5](#_Toc221191434)

[Section S5. Evolution process of in-plane guided mode to out-plane OAM mode	7](#_Toc221191435)

[Section S6. The mapping relationship between waveguide modes and structured light beams (OAM, LP, and vector modes)	8](#_Toc221191436)

[Section S7. Phase shifter calibration and cross-talk mitigation	11](#_Toc221191437)

[Section S8. Device fabrication process	12](#_Toc221191438)

[Section S9. Experiment results of the other channels	13](#_Toc221191439)

[Section S10. Experiment results of OAM mode demodulation	14](#_Toc221191440)

[Section S11. Experiment results of vector beams on HOPS	14](#_Toc221191441)

[Section S12. Experiment results of typical vector beams on 4D Hilbert space	15](#_Toc221191442)

[Section S13. Polarization characterization and reconstruction	15](#_Toc221191443)

[Section S14. Numerical analysis of the fabrication tolerance	16](#_Toc221191444)

[Section S15. Experimental Configuration	17](#_Toc221191445)

[Section S16. A comparison of on-chip structured light beam generators	18](#_Toc221191446)

[Section S17. Vector quality factor (VQF)	18](#_Toc221191447)

[Section S18. Efficiency and purity trade-off	19](#_Toc221191448)

[Section S19. Broadband generation of vector beam	19](#_Toc221191449)

[Section S20. Thermo-optic (TO) phase shifter’s response speed	21](#_Toc221191450)

[References:	21](#_Toc221191451)

Section S1. The mapping order selection

**Fig. S1. Illustration of holographic method producing superposed fork grating. (a)** Phase and intensity profiles of two waveguide modes (TE_0_ and TE_1_) before coupling to the grating. **(b)** Synthesis process of the initial holographic grating employed in this study. **(c)** Schematic illustration of an alternative grating synthesis sequence, representing one randomly selected pathway among numerous feasible synthesis approaches.

Figure S1(a) shows the simulated electric field and phase distributions of the TE_0_ and TE_1_ modes within the silicon waveguide, captured prior to their injection into the meta-waveguide. The fundamental TE_0_ mode exhibits a well-confined elliptical profile with a spatially uniform phase across the waveguide cross-section. In contrast, the first-order TE_1_ mode is characterized by a distinctive dual-lobe intensity distribution, where the two lobes exhibit an anti-phase relationship (a π phase shift).

Regarding the selection of grating combinations, a more detailed explanation is provided in the figure below. In practice, the superposition of spiral phases and waveguide modes in grating synthesis can be arbitrarily configured, as illustrated in Fig. S1(b, c).

In our device design, we adopted the combination sequence shown in Fig. S1(b): Grating 0 is formed by superimposing a spiral phase with orbital angular momentum (OAM) of +1 onto the TE₁ waveguide mode, while Grating 1 is generated by superimposing an OAM of +2 onto the TE₀ mode. These two resulting gratings, Grating 0 and Grating 1, are then combined to form Grating A. Grating B is obtained by rotating Grating A, and the final holographic grating is synthesized by performing a logical OR operation between Gratings A and B.

Figure S1(b) The synthesis process of the initial holographic grating employed in this study is illustrated from left to right. The first column depicts the phase profile of the orbital angular momentum (OAM) beam, while the second column shows the phase distribution of the guided mode propagating in the silicon waveguide. The third column presents the binary fork-shaped grating obtained by superimposing the previous two phase profiles and applying binarization. In the fourth column, the grating field distribution is generated by combining the upper half of the first grating and the lower half of the second grating from the third column. Subsequently, the final eight-channel holographic grating is formed by superimposing the grating from the fourth column with its 90° rotated counterpart.

Section S2. Effective index and propagation constant

In general, distinct guided modes (e.g., TE_0_ vs. TE_1_) exhibit different propagation constants due to their unique transverse field distributions, as dictated by waveguide dispersion relations. However, in our design, we tailored the strip waveguide’s geometric parameters to achieve near-identical propagation constants for TE_0_ and TE_1_ modes.

**Fig. S2. n_eff_ (a) and propagation constants (b) of TE_0_ and TE_1_ modes.**

Specifically, we simulated the effective refractive indices n_eff_ of TE_0_ and TE_1_ modes as a function of waveguide width. Our results show that when the strip waveguide width is optimized to *W* = 3.6 μm, the *n*_eff_ value of TE_0_ and TE_1_ modes converge to nearly the same value. Using the propagation constant formula $\beta_{eff}$ = *n*_eff_×2π×*λ*^-1^, this shared *n*_eff_ translates to a common $\beta_{eff}$ ≈ 11.4 μm^-1^ for both modes. Importantly, this convergence is specific to TE_0_ and TE_1_ modes: TE_0_ and TM_0_ modes (with orthogonal polarization) still maintain distinct *n*_eff_ and $\beta_{eff}$values under the same waveguide width, consistent with fundamental dispersion characteristics of guided waves.

This intentional optimization of waveguide width ensures phase matching between TE_0_ and TE_1_ modes during their propagation and subsequent conversion to OAM states.

Section S3. Details of simulation and optimization process

The 3D FDTD simulations assisted with improved direct-binary-search (DBS) algorithm are performed to design optimum pattern in Fig. S3(a). We first toggle the logical state of one pixel to calculate the initial FOM. Then, the pixel state is retained once the FOM is improved, if not, the pixel will be toggled to the original state. Our DBS optimization follows an iterative pixel-wise update process: each iteration traverses all meta-unit pixels to adjust their binary states (silicon present/absent) based on whether the change improves the Figure of Merit (FOM, defined as average mode purity). The optimization is deemed convergent when the FOM exhibits no significant improvement (specifically, a relative increase of <5%) over three consecutive iterations. This condition balances optimization efficiency and performance—ensuring we avoid over-optimization (which could reduce fabrication tolerance) while achieving sufficiently high mode purity. It takes about 168 h to obtain the optimized pixel array of the meta-waveguide after two rounds of iteration, on the workstation with a twenty-core central processing unit (Intel Core i9-10900X). Figure S3(b) indicates the 2D pixel array pattern and calculated FOM of initial structure, first iteration and second iteration, respectively. As can be seen, the FOM increases from the 0.75 to 0.84.

**Fig. S3. Schematic of simulation and optimization process. (a)** Flow chart for the optimization process. **(b)** The optimized 2D pixel array pattern and corresponding FOM.

Section S4. Simulation results of initial and optimized structures

Figure S4 characterizes the performance of the OAM generation. Fig. S4(a) and Fig. S4(b) present the scattering efficiency and beam purity as functions of etch depth, respectively. Based on these correlations, we selected an etch depth of 130 nm to achieve an optimal balance between scattering efficiency and beam purity. The wavelength-dependent beam purity of the holographic grating and the pre-optimized grating are shown in Figures Fig. S4(d) and Fig. S4(e), respectively. Comparison of these results indicates that discretizing the holographic grating maintains nearly consistent beam purity across the 1500–1600 nm wavelength range. As demonstrated in Figures Fig. S4(c) and Fig. S4(f), the inversely optimized grating exhibits enhanced scattering efficiency and improved beam purity under broadband operation compared to the initial design. These results confirm the efficacy of our inverse design strategy for optimizing optical field properties.

**Fig. S4. Simulated scattering efficiency and purity.** **(a)** Scattering efficiency and **(b)** mode purity versus etch depth ***h*** of the initial photonic-crystal-like structure for OAM generation. **(c)** Wavelength-dependent scattering efficiency of the final optimized device. **(d)** Mode purity versus wavelength of the holographic grating at 130 nm etch depth. **(e)** Mode purity versus wavelength of the initially forward-designed composite grating. **(f)** Wavelength-dependent mode purity of the final optimized device.

Figure S5(a) presents the electric field intensity, phase distribution, and collinear interferograms of the eight OAM modes generated by the initial PhC-like structure array before optimization at 1550 nm. The field intensity profiles reveal incomplete annular structures, indicating deviations from ideal vortex beams. Notably, the phase singularities of the OAM_-2_ and OAM_+2_ modes appear at two distinct locations, exhibiting discontinuous phase features. Similar characteristics are observed for all eight OAM modes in Fig. S5(b). These results confirm that the initial structure exhibits significantly degraded vortex beam properties compared to the optimized design, underscoring the effectiveness of the inverse design approach in achieving high-purity OAM generation.

We employed the overlap integral method to quantify the mode purity of the generated beams, a approach consistent with the power coupling efficiency principle (i.e., characterizing the power conversion efficiency of the obtained field to the target mode). The overlap integral for mode purity calculation is defined based on the complex amplitudes of the obtained field and the ideal target mode, following the expression [1]:

$$P=\frac{|\int E_{0}^{*}(x,y,z)E_{t}(x,y,z)dxdydz|^{2}}{\int|E_{0}^{*}(x,y,z)|^{2}dxdydz\int|E_{t}(x,y,z)|^{2}dxdydz}$$

where *E*_0_(*x, y, z*) and *E_t_*(*x, y, z*) represent the complex amplitudes of the obtained mode and the ideal target mode (e.g., the designed OAM in this work), respectively. In our process, we first performed spatial alignment of the obtained field relative to the target mode: adjusting the scaling factor to match the beam waist of the obtained field with that of the target mode, and centering the geometric center of the obtained field to coincide with the target mode’s center. We then calculated the overlap integral under different scaling/centering parameters and selected the maximum integral value as the final basis for mode purity—this ensures the purity result reflects the optimal matching degree between the obtained field and the target mode.

**Fig. S5. Simulated intensity profiles, phase distributions, and interferograms for (a) 2D holographic grating and (b) 2D initial PhC-like structure.**

Section S5. Evolution process of in-plane guided mode to out-plane OAM mode

To investigate the evolution process of in-plane guided mode to out-plane OAM mode, we simulate the electrical field distributions of the yellow square region (width = 6 μm) in Fig. S6 (including waveguide region and grating region) at different height (*z* = 0.2, 0.4, 0.6, 0.8, 1.0 μm, far field). *z* = 0 represents the bottom of the silicon core layer. Figure S6 indicates the real part distributions of the electric field components of 8 OAM modes for 2D meta-waveguide (*y*-Pol. OAM_-2_, *x*-Pol. OAM_-2_, *y*-Pol. OAM_+2_, *x*-Pol. OAM_+2_, *y*-Pol. OAM_-1_, *x*-Pol. OAM_-1_, *y*-Pol. OAM_+1_ and *x*-Pol. OAM_+1_), respectively. We can observe the mode evolution from the guided mode to free space mode based on the electric field distributions of different height.

**Fig. S6. Simulation results for the evolution process of in-plane guided modes (TE_0_ and TE_1_ ) to out-plane OAM modes.**

Section S6. The mapping relationship between waveguide modes and structured light beams (OAM, LP, and vector modes)

Based on the OAM mode basis enabled by the proposed 2D meta-waveguide, on-chip multiple guided modes can be further mapped to more complex structured beams, including arbitrarily polarized OAM modes on conventional Poincaré spheres (PSs), linearly-polarized (LP) modes on OAM PSs, and cylindrical vector (CV) modes on higher-order Poincaré spheres (HOPSs). By controlling the amplitude and phase of the guided modes, arbitrary structured light beams could be generated by the proposed device. For example, LP modes on OAM orbit could be generated by combining two OAM modes with same polarization and opposite topological charges. The vector modes on HOPSs could be generated by combining left- and right-circularly polarized OAM modes with both opposite spin polarizations and topological charges.

Figure S8 presents the FDTD-simulated intensity and polarization-resolved (horizontal and vertical) profiles of four representative optical fields on the equatorial plane (*θ* = π/2) of the +1^st^-order HOPS shown in Fig. 3c. Similarly, Fig. S9 provides the corresponding results for the +2^nd^-order HOPS. Furthermore, Figure S10 illustrates the 4D superposition states synthesized by combining the -1^st^-order HOPS state (*θ* = π/2, *φ* = π) and the -2^nd^-order HOPS state (*θ* = π/2, *φ* = π/2); we also provide the horizontal and vertical field distributions on the equatorial plane for these states.

**Fig. S7. Decompositions of (a) various polarization states on polarization PS and (b) LP modes on OAM PS in terms of orthogonal linear polarization states and** **orthogonal OAM modes, respectively. (c) Decomposition of vector modes on HOPSs in terms of orthogonal OAM modes. The color change indicates the relative phase of the components. Arrows indicate polarization direction.**

**Table S1.** Mapping of 2D PIC guides modes to OAM, LP, and vector modes

| PIC modes | | Port 2  +*x* TE_0_ | Port 1  -*x* TE_0_ | Port 2  +*x* TE_1_ | Port 1  -*x* TE_1_ | Port 4  +*y* TE_0_ | Port 3  -*y* TE_0_ | Port 4  +*y* TE_1_ | Port 3  -*y* TE_1_ |
| --- | --- | --- | --- | --- | --- | --- | --- | --- | --- |
| OAM modes | | *y*-Pol.  OAM_+2_ | *y*-Pol.  OAM-_2_ | *y*-Pol.  OAM_+1_ | *y*-Pol.  OAM_-1_ | *x*-Pol.  OAM_+2_ | *x*-Pol.  OAM-_2_ | *x*-Pol.  OAM_+1_ | *x*-Pol.  OAM_-1_ |
| LP modes on OAM PSs | LP^o^_21_*_y_* | 1 | 1 |  |  |  |  |  |  |
|  | LP^e^_21_*_y_* | 1 | -1 |  |  |  |  |  |  |
|  | LP^o^_11_*_y_* |  |  | 1 | 1 |  |  |  |  |
|  | LP^e^_11_*_y_* |  |  | 1 | -1 |  |  |  |  |
|  | LP^e^_21_*_x_* |  |  |  |  | 1 | 1 |  |  |
|  | LP^o^_21_*_x_* |  |  |  |  | 1 | -1 |  |  |
|  | LP^e^_11_*_x_* |  |  |  |  |  |  | 1 | 1 |
|  | LP^o^_11_*_x_* |  |  |  |  |  |  | 1 | -1 |
| Vector modes on HOPSs | *m*=1, *φ*_0_=0 |  |  | -1 | 1 |  |  | -1 | 1 |
|  | *m*=1, *φ*_0_=π/4 |  |  | -i | 1 |  |  | -i | 1 |
|  | *m*=1, *φ*_0_=π/2 |  |  | 1 | 1 |  |  | 1 | 1 |
|  | *m*=1, *φ*_0_=3π/4 |  |  | 1 | -i |  |  | 1 | -i |
|  | *m*=2, *φ*_0_=0 | i | -i |  |  | 1 | 1 |  |  |
|  | *m*=2, *φ*_0_=π/4 | -i | 1 |  |  | -1 | i |  |  |
|  | *m*=2, *φ*_0_=π/2 | 1 | 1 |  |  | -i | i |  |  |
|  | *m*=2, *φ*_0_=3π/4 | i | 1 |  |  | 1 | i |  |  |
| Vector modes on HOPSs | *m*=-1, *φ*_0_=0 |  |  | 1 | -1 |  |  | -1 | 1 |
|  | *m*=-1, *φ*_0_=π/4 |  |  | 1 | -i |  |  | -1 | i |
|  | *m*=-1, *φ*_0_=π/2 |  |  | 1 | 1 |  |  | -1 | -1 |
|  | *m*=-1, *φ*_0_=3π/4 |  |  | 1 | i |  |  | -1 | -i |
|  | *m*=-2, *φ*_0_=0 | -i | i |  |  | 1 | 1 |  |  |
|  | *m*=-2, *φ*_0_=π/4 | -i | -1 |  |  | 1 | i |  |  |
|  | *m*=-2, *φ*_0_=π/2 | -i | -i |  |  | 1 | -1 |  |  |
|  | *m*=-2, *φ*_0_=3π/4 | -i | 1 |  |  | 1 | -i |  |  |

**Fig. S8. Simulation results for the generation of first-order CV beams mapped on HOPS.**

**Fig. S9. Simulation results for the generation of second-order CV beams mapped on HOPS.**

**Fig. S10. Simulation results for the generation of 4D superposition states.**

Section S7. Phase shifter calibration and cross-talk mitigation

1. Amplitude calibration first

Since amplitude modulation also introduces an additional phase shift, we first calibrate the channel amplitude and then the phase shift. We gradually adjust the voltage applied to the thermo-optic phase shifters of one channel (keeping the other channel’s voltage fixed at the nominal value). As voltage changes, the thermal-optic effect modulates the waveguide’s refractive index, which in turn slightly alters the mode’s transmission efficiency (amplitude) via an MZI. We record the output amplitude of the tuned channel at each voltage step, plotting a “voltage-amplitude” curve. This allows us to identify the voltage range that stabilizes the amplitude to the target value.

2. Calibration Procedure for Phase Shifters (Addressing Initial Phase Offset)

Despite the highly symmetric design of our chip architecture, inherent fabrication errors (e.g., minor variations in waveguide width or film thickness) introduce small initial phase offsets between channels. To characterize and compensate for these offsets, we developed a two-step calibration workflow, which directly links applied voltage to phase shift and eliminates initial discrepancies:

Step 1: Relative Phase Calibration for Counter-Propagating Channels

For the two counter-propagating channels in each module, we first generate polarization-matched OAM_±1_ modes (e.g., both in the *x*-polarization state). These two modes interfere at the output detector, forming a distinct interference pattern whose fringe spacing and orientation are sensitive to the relative phase between the channels. By gradually adjusting the voltage applied to the thermo-optic phase shifters of one channel, we observe real-time changes in the interference fringes: when the fringes return to the “reference pattern” (simulated for zero relative phase), we record the corresponding voltage-phase relationship, and repeat this process 4 times for each pair of counter-propagating channels.

Step 2: Polarization-Orthogonal Channel Calibration

For the two vertically orthogonal channels (*x*-polarization vs. *y*-polarization), we generate OAM modes with orthogonal polarizations (e.g., *x*-polarized OAM_+1_ and *y*-polarized OAM_-1_) and analyze their polarization state at the output using a polarization analyzer. The initial phase offset between these channels manifests as a deviation of the measured polarization ellipse from the ideal “linear polarization” reference. By tuning the phase shifters of the *y*-polarization channel until the analyzer detects the ideal linear polarization state, we can establish the voltage-phase calibration curve for this channel.

Through this two-step process, we not only characterize the initial phase offset of each of the 16 phase shifters but also build a lookup table that maps target phase shifts to precise applied voltages—enabling accurate tuning to any desired state on the complex Poincaré sphere.

Section S8. Device fabrication process

As indicates in Fig. S11, the two optimized ultra-compact OAM emitters based on PhC-like SWG structures were fabricated and experimentally demonstrated on standard silicon-on-insulator (SOI) wafer. We use an electron beam lithography system (Vistec EBPG 5000 Plus) to form the optimized pattern on SOI platform with a 220 nm thick top silicon layer and an inductively coupled plasma etcher (Plasma lab System100) to transfer the mask to the silicon device layer. The pixel-array is approximately etched up to 130 nm by setting the proper etching time, while the waveguide outlines are fully etched down 220 nm to the buried oxide. A 2 μm thick SiO_2_ layer is deposited on the silicon layer by plasma-enhanced chemical vapor deposition (PlasmaPro 800 Stratum PECVD).

**Fig. S11. Illustration of the device fabrication process of the devices (spin coating, electron-beam lithography, inductively coupled plasma, photoresist removal, and PECVD upper SiO_2_ cladding).**

Section S9. Experiment results of the other channels

**Fig. S12. Measured additional results for broadband vortex beam generation with the meta-waveguide.**

Figure S12 shows the measured far-field intensity, coaxial interferograms, optical field demodulated via a SLM of six OAM modes for 2D meta-waveguide (*y*-Pol. OAM_-2_, *y*-Pol. OAM_+2_, *x*-Pol. OAM_+2_, *y*-Pol. OAM_-1_, *x*-Pol. OAM_-1_, and *x*-Pol. OAM_+1_) at 1530, 1550 and 1565 nm. Doughnut shaped intensity profiles and spiral interference patterns verify the helical phase front of the generated OAM modes. Topological charge order can be recognized by the number of spiral arms and the spiral direction in the interferograms. Gaussian-type demodulated optical fields reveal good performance of the generated OAM modes. Figure S13 illustrates that as the polarization angle rotates from 0° to 90°, the optical power of the *x*-Pol. OAM_-2_ mode (and its corresponding field after SLM demodulation) gradually decreases from its maximum to its minimum, whereas the power of the *y*-Pol. OAM_+1_ mode exhibits the opposite trend, increasing accordingly.

**Fig. S13. Measured results for the verification of the emission light polarization state.**

Section S10. Experiment results of OAM mode demodulation

As shown in Fig. S14, the emitted OAM modes of the fabricated 1D meta-waveguide can be demodulated to a bright spot at the beam center (Gaussian-like beam) when meeting an inverse phase pattern, while remains the doughnut field profile with another topological charge in the case of non-corresponding demodulation phase pattern. Meanwhile, the orthogonal component of the detecting mode appears weak light. Similarly, the demodulated performance of 2D meta-waveguide is clearly characterized in Fig. S14, with corresponding Gaussian-like points in the diagonal elements.

**Fig. S14. Measured results for eight OAM modes demodulation.**

Section S11. Experiment results of vector beams on HOPS

**Fig. S15. Fully tunable synthesis of vector beams on the equator of +1^st^-order HOPS.**

Figure S15 presents four additional representative optical fields on the equator of the +1^st^-order HOPS, corresponding to positions 2, 4, 6, and 8 as labeled in the figure. For each position, the following field characteristics are displayed: the original field without a polarizer, the field transmitted through polarizers at four distinct orientations, and the left- and right-handed circularly polarized components obtained after decomposition using a quarter-wave plate. These results further demonstrate the device’s capability for continuous and arbitrary tuning along the latitudinal direction of the HOPS. The corresponding experimental demonstration is provided in Supplementary Video S2.

Section S12. Experiment results of typical vector beams on 4D Hilbert space

The figure below demonstrates the generation of optical fields within a four-dimensional (4D) Hilbert space through the strategic superposition of various basis vectors. Specifically, Beam 1 is synthesized from the combination of basis vectors -1^st^-order HOPS (*θ* = π/2, *φ* = π) and -2^nd^-order HOPS (*θ* = π/2, *φ* = π/2), whereas Beam 2 is derived from -1^st^-order HOPS (*θ* = π/2, *φ* = 0) and -2^nd^-order HOPS (*θ* = π/2, *φ* = π/2). By analyzing the individual polarization components, we characterize the underlying polarization topology and distribution features. These results collectively underscore the device's capability for the flexible and high-fidelity synthesis of structured light across a high-dimensional Hilbert space.

**Fig. S16. Typical vector beams on 4D Hilbert space.**

Section S13. Polarization characterization and reconstruction

The spatially varying polarization distributions of the generated vector beams were reconstructed by measuring the Stokes parameters (*S*_0_, *S*_1_, *S*_2,_ *S*_3_). Specifically, six intensity components were recorded: *I*_R_, *I*_L_, *I*_0_, *I*_45_, *I*_90_ and *I*_135_. Here, *I*_R_ and *I*_L_ denote the intensities of the right- and left-circularly polarized components, respectively, acquired using a quarter-wave plate (QWP) and a linear polarizer. The remaining four intensities *I*_0_, *I*_45_, *I*_90_ and *I*_135_ represent the linearly polarized components obtained by orienting a linear polarizer at 0°, 45°, 90°, and 135° relative to the horizontal axis. The Stokes parameters were calculated as follows[2]

$$\begin{matrix} S_{0}=I_{0}+I_{90} \\ S_{1}=I_{0}-I_{90} \\ S_{2}=I_{45}-I_{135} \\ S_{3}=I_{R}-I_{L} \end{matrix}$$

From these Stokes parameters, the local polarization state at each pixel—geometrically represented by the polarization ellipse—can be rigorously determined. Specifically, the polarization orientation angle *ψ* (azimuth of the major axis relative to the horizontal) and the ellipticity angle *χ* are calculated using the following relations [2]

$$\psi=\frac{1}{2}\arctan(\frac{S_{2}}{S_{1}}),\chi=\frac{1}{2}\arcsin(\frac{S_{3}}{S_{0}})$$

Here, *ψ* indicates the rotation of the polarization axis, while *χ* characterizes the degree of ellipticity (with *χ* = 0 representing linear polarization and *χ*= ±π/4 representing left/right circular polarization). By mapping these parameters across the beam profile, we successfully reconstructed the polarization state distribution of the light field (as shown in the last column in Fig. 7 of the main text).

Section S14. Numerical analysis of the fabrication tolerance

To investigate the fabrication tolerance of the OAM emitters, we here change the diameter range and the depth range of etching hole and simulate the performance of OAM modes. Figure S17 presents simulations of the average purity of 8 modes in the 2D meta-waveguide, plotted against radius/etching depth variations at the 1550 nm center wavelength. Within a diameter range of 70–110 nm (ideal target: 90 nm), the average mode purity fluctuates by 0.01-0.04 , as shown in Fig. S17(a), leading to increased mode crosstalk. Within a depth range of 110–150 nm (ideal target: 130 nm), the average mode purity fluctuation is only 0.01–0.015 , as shown in Fig. S17(b), indicating minimal impact on mode crosstalk. These results indicate that the devices maintain consistent performance under such fabrication-induced variations. To further improve fabrication tolerance, an inverse design method called robust adjoint method could be used to design fabrication-tolerant digital nanophotonic devices. In the proposed robust adjoint method, multiple patterns with identical pixel distribution but different pixel sizes/etch depth can be taken into account, ensuring that the optimized designs maintain high performance when the pixels suffer diameter variations.

**Fig. S17. Numerical analysis of the fabrication tolerance.** Simulated results for 2D meta-waveguide. **(a)** Numerical analysis of the diameter of the hole. **(b)** Numerical analysis of the depth of the hole.

Section S15. Experimental Configuration

**Fig. S18. Experimental setup.** PC, polarization controller; VOA, variable optical attenuator; Col., collimator; Pol., polarizer; HWP, half-wave plate; BS, beam splitter; SLM: spatial light modulator.

The measurement setup shown in Fig. S18 is used to characterize the performance of OAM emitters. The light from a tunable light source with the wavelength from 1530 to 1565 nm was split into two branches by a 3-dB coupler. One branch was attenuated to the appropriate power and collimated to produce the reference Gaussian beam. A polarizer and a half wave plate (HWP) were used to ensure a consistent polarization between the emission beam and the reference beam. The other branch passed through polarization controller (PC) and was coupled into the chip by a lensed fiber. The generated polarization and order diversity OAM mode was collimated by an objective lens (10$\text{×}$, NA= 0.26). The beam size and polarization can be adjusted by polarizer, half wave plate (HWP) and lens. The intensity profiles of the generated OAM modes and their interference patterns with the reference Gaussian beams were monitored with an infrared CCD camera (Xenics, Bobcat-640-GigE). The other branch of OAM separated by the beam splitter was demodulated to Gaussian-like beam by a spatial light modulator (SLM, Meadowlark Optics E19$\text{×}$12). One branch of the demodulated beam was captured by the infrared CCD camera to get the field intensity. The other branch was coupled into a single mode fiber connected with the power meter to measure the optical power.

Section S16. A comparison of on-chip structured light beam generators

**Table S2. A comparison of on-chip structured light beam generators**

Section S17. Vector quality factor (VQF)

We calculated the vector quality factor (VQF) of the five vector beams in Fig. 6 via a basis-independent method[3] using Stokes parameters. In this approach, we first measured six intensities to calculate Stokes parameters: *I*_R_, *I*_L_, *I*_0_, *I*_45_, *I*_90_ and *I*_135_. *I*_R_ and *I*_L_ are the intensities corresponding to the right-circularly polarized and left-circularly polarized components of the beam, respectively, acquired using a quarter-wave plate (QWP) oriented at specific angles in combination with a linear polarizer. The remaining four intensities—namely *I*_0_, *I*_45_, *I*_90_ and *I*_135_—are obtained by passing the beam through a linear polarizer rotated to 0°, 45°, 90°, and 135° relative to the horizontal axis, respectively, representing the linearly polarized components along these specific orientations. The Stokes parameters can be calculated as equation (2). The VQF was then determined by [3]

$$V=\sqrt{1-(S_{1}^{2}+S_{2}^{2}+S_{3}^{2})/S_{0}^{2}}$$

where $S_{i}=\int S_{i}^{'}(\mathbf{r})\text{d}\mathbf{r}$ are global Stokes parameters integrated over the transverse plane. With this method, we can calculate the vector quality factor of 0.9757, 0.9880, 0.9444, and 0.9971 for the vector beams in Fig. 6b, respectively.

We note that the VQF values here are higher than the mode purity values. This discrepancy arises from their distinct evaluation focuses: VQF primarily characterizes the polarization distribution of the optical field, with its calculation centered on the "vector" polarization properties of the field [4,5]. In contrast, OAM mode purity is calculated using the overlap integral method, which focuses on matching the amplitude and phase distributions of the measured mode to the ideal OAM mode profile. This phenomenon is consistent with observations in the cited literature [6], where similarly high VQF values were reported alongside relatively low OAM mode purity—further confirming that the two metrics describe different aspects of the optical field and thus do not follow a direct positive correlation.

Section S18. Efficiency and purity trade-off

A trade-off between purity and efficiency does indeed exist. Optimizing the grating for higher beam purity generally leads to a reduction in diffraction efficiency, while designs targeting higher efficiency tend to compromise purity. To mitigate this constraint, a larger grating area can be employed together with inverse design methods, which help achieve higher diffraction efficiency while maintaining beam purity.

**Fig. S19. Impact of waveguide dimensions. a**. Diffraction Efficiency versus Waveguide Size. **b**. Beam Purity versus Waveguide Size

We have included plots showing the variation in diffraction efficiency and purity for holographic gratings of different sizes. Note that these structures refer to continuous holographic gratings, rather than discretized or inversely-optimized configurations. As shown in Fig. S19(a), diffraction efficiency increases significantly with grating size, reaching nearly 50% for a grating area of 12 μm × 12 μm. Figure S19(b) illustrates the corresponding trend in purity: although purity tends to decrease as the grating area expands, it can be improved by discretizing the holographic grating and applying inverse design to optimize the meta-waveguide structure. This approach effectively transforms the purity–efficiency trade-off into a structure–efficiency balancing challenge, which can be systematically addressed through advanced design strategies.

Section S19. Broadband generation of vector beam

Regarding the variation in the radiation angle of the emitted light due to changes in wavelength, we provide the following more detailed description:

**1. Angular Deviation Characterization**

When the operating wavelength deviates from the design value, the guided waves incident on the diffraction grating from different directions exhibit distinct angular shifts. As shown in our simulations—taking the light incident from the right-side waveguide as an example—the optical field monitored at a distance of 4 μm above the chip surface shifts in the Y-direction as the wavelength varies from 1500 nm to 1600 nm. Within this wavelength range, the maximum angular shift remains within ±8°, which has a negligible impact on the overall field distribution. These results align well with the behavior reported in Ref. [7]. Furthermore, the simulated field displacement between 4 μm and 8 μm above the chip is only about 1 μm, as shown in Fig. S20 (b, c). At such propagation distances, this minor shift does not significantly affect the synthesis of the output beam.

**Fig. S20. Beam deflection and shift induced by wavelength variation. (a)** Variation of deflection angle with wavelength. **(b)** Optical field at 4 μm from chip surface. **(c)** Optical field at 8 μm from chip surface. With a pixel resolution of 239×239 over an actual area of 12 μm, the measured displacement from 4 μm to 8 μm corresponds to (20/239)×14 ≈ 1.00 μm.

**2. Beam Synthesis and Experimental Observation**

**Fig. S21. Vector beam profiles across 1530–1600 nm spectrum and corresponding polarization-resolved measurements.**

In practice, we are able to synthesize vector beams over a broad wavelength range in our experiments. This is achieved by configuring the optical system such that the infrared CCD camera captures the optical field within a few micrometers from the chip surface. At this observation plane, beam steering caused by wavelength drift is effectively mitigated, allowing stable superposition and synthesis of vector beams. Moreover, the output from all channels remains centered at the grating region. The corresponding experimental demonstration is provided in Supplementary Video S1.

From an optical path perspective, we effectively map the image plane to an object-side plane very close to the chip’s emission surface. The angular deviation below 8° induced by wavelength variation is managed through the optical system, producing minimal displacement of the spot on the CCD. The figure below demonstrates successful vector beam synthesis across wavelengths from 1530 nm to 1600 nm. Results confirm that vector beams can be synthesized across the entire C+L band and properly decomposed using a polarizer.

In summary, by appropriately designing the optical system and transmission path, the receiving plane can be effectively mapped to the emission plane, thereby managing for diffraction angle variations induced by wavelength drift.

Section S20. Thermo-optic (TO) phase shifter’s response speed

**Fig. S22. Respond speed of the TO phase shifter.**

We have incorporated experimental characterization of the thermo-optic phase shifter's temporal response. We evaluated the transient performance of TiN heaters under square-wave electrical modulation at 5 kHz. The measured rise and fall times were determined to be 21.4 μs and 22 μs, respectively. This performance is fundamentally constrained by the thermal dissipation characteristics inherent to the TiN heater and SiO₂/Si substrate structure. We acknowledge that implementing alternative approaches such as PN junction phase shifters or lithium-niobate-on-insulator (LNOI) modulators would enable switching performance in the nanosecond regime, representing a substantial improvement over the current thermo-optic implementation.

References:

[1] Zhou, N. et al. Ultra-compact broadband polarization diversity orbital angular momentum generator with 3.6 × 3.6 µm2 footprint. *Science Advances* **5**, eaau9593 (2019).

[2] Naidoo, D. et al. Controlled generation of higher-order Poincaré sphere beams from a laser. *Nature Photonics* **10**, 327-332 (2016).

[3] Isaac, N. et al. Revealing the invariance of vectorial structured light in complex media. *Nature Photonics* **16**, 538-546 (2022).

[4] Ndagano, B. et al. Beam quality measure for vector beams. *Optics Letters* **41**, 3407-3410 (2016).

[5] Liang, Y. et al. Coherent detector for the non-separability measurement of vectorial structured light. *Light: Science & Applications* **14**, 343 (2025).

[6] Zhao, W. et al. All-on-chip reconfigurable generation of scalar and vectorial orbital angular momentum beams. *Light: Science & Applications* **14**, 227 (2025).

[7] Zhou, N. et al. Generating and synthesizing ultrabroadband twisted light using a compact silicon chip. *Optics Letters* **43**, 3140-3143 (2018).
